# Tadalafil, a long acting phosphodiesterase inhibitor, promotes bone marrow stem cell survival and their homing into ischemic myocardium for cardiac repair

**DOI:** 10.14814/phy2.13480

**Published:** 2017-11-15

**Authors:** Ibrahim Elmadbouh, Muhammad Ashraf

**Affiliations:** ^1^ Department of Emergency Medicine Davis Heart and Lung Research Institute Wexner Medical Center Ohio State University Columbus Ohio; ^2^ Faculty of Medicine Menoufia University Shebin Elkom Egypt

**Keywords:** ^111^In‐oxine imaging, myelo‐ablation, pharmacological preconditioning, stem cell survival

## Abstract

The aim was to evaluate the tadalafil‐mediated effects at molecular level on bone marrow‐derived mesenchymal stem cells (MSCs) survival and their homing into the infarcted hearts to promote cardiac repair and improve function. MSCs were pretreated in vitro with inhibitors of PKG, MAPK, FasL, nitric oxide synthase (NOS) (L‐NAME), CXCR4 (AMD3100), or miR‐21 inhibitors (+/−luciferase construction +/−Fas) prior to tadalafil treatment for 2 h. These MSCs were then subjected to H_2_O_2_ stress to assess their injury. Rats were subjected to acute myocardial infarction (AMI), and then followed by injection of saline or 1.5 x 10^6^ MSCs‐treated ± tadalafil into infarcted and peri‐infarcted area. In another group, AMI was performed in 1‐month post‐myelo‐ablated rats and were injected intraperitoneally (IP) with tadalafil ± AMD3100 or L‐NAME for 5 days. Also, in another group, AMI mice were treated with IP ± tadalafil before intravenous injection with ^111^In‐oxine‐MSCs followed by CT/SPECT imaging to locate mobilized MSCs. Cardiac function was assessed by echocardiography. MSCs and heart extracts were analyzed by molecular bioassays. Tadalafil‐treated MSCs had higher expression of cGMP, NOS, SDF‐1*α*, p‐VASP, p‐Erk1/2, p‐STAT3, p‐Akt, PKG1 and Bcl‐xl; expression of these molecules was reduced with PKG1, MAPK, NOS or FasL inhibitors. Tadalafil inhibited apoptosis through increased miR‐21 expression and improved cell survival by inhibiting Fas (restored by PKG1, MAPK or miR‐21 inhibitors). In vivo, heart function, grafted cell survival, MSCs mobilization and homing were improved in tadalafil‐treated AMI animals versus controls. Conclusions: Tadalafil prolonged MSCs survival via up‐regulation of miR‐21 dependent suppression of Fas, and increased MSCs mobilization and their homing into infarcted myocardium resulting in improved cardiac repair and function.

## Introduction

Although pump performance in hearts with regional myocardial ischemia is improved after cell‐based therapy, low cell retention and survival limit the long‐term regeneration by stem cells (Elmadbouh et al. 2007). Our previous findings that treatment of donor stem cells with – transient hypoxia (ischemic preconditioning) (Kim et al. 2009), agents including diazoxide (pharmacological preconditioning) (Niagara et al. 2007), or vascular endothelial growth factor 2 (VEGF‐2) and insulin‐like growth factor‐1 (IGF‐1) (Haider et al. 2008a), stromal derived factor‐1‐alpha (SDF‐1*α*) (genetic preconditioning) (Elmadbouh et al. 2007, 2011) – have been proposed as effective methods to improve grafted cell survival in the infarcted heart (Pasha et al. 2008). Nevertheless, continuing efforts to develop newer approaches and safer strategies to improve the lasting regenerative potential of stem cells are needed to refine the best approaches to assure their clinical utility.

A promising and clinically relevant preconditioning agent such as the phosphodiesterase‐5 (PDE‐5) inhibitor (tadalafil) offers the prospect of cytoprotection for donor stem cells. Although we have shown that tadalafil promotes cardioprotection in vitro (Kumar and Ashraf 2015) and after acute myocardial infarction (AMI) in vivo through its effects on cyclic guanosine monophosphate (cGMP)‐protein kinase G (PKG1) (Ahmad et al. 2009; Haider et al. 2010a; Li et al. 2012); the mechanisms underlying tadalafil's effects on stem cell survival in vivo are incompletely understood. In this study, we attempt to define the novel pathways by which tadalafil treatment leads to cardioprotection.

On possible mechanism by which tadalafil might afford cardioprotection is that it may modulate miR21‐dependent suppression of Fas (CD95, tumor necrosis factor (TNF) receptor super family member). The interactions between Fas and Fas ligand (FasL, CD178) are recognized to induce apoptosis (Sayed et al. 2010) in cells and in the heart during failure and ischemia (Lee et al. 2003). Our previous study showed that miR‐21 was a key anti‐apoptotic regulator in preconditioning of donor stem cells (Haider et al. 2010b) through miR‐21‐dependent suppression of FasL (Sayed et al. 2010).

PDE‐5 inhibitors, including tadalafil, have been approved for clinical use to reverse erectile dysfunction (Foresta et al. 2006) and reduce pulmonary arterial hypertension (Ghofrani et al. 2004). These agents are also known to increase circulating endothelial progenitor cells (EPC) and CXCR4 expression (Foresta et al. 2010). Therefore, tadalafil might be expected to enhance the cell survival of transplanted MSCs in vivo, and might even accelerate cardiac stem cell homing to injured myocardium. Such a finding could serve as a pivotal element to advance cell therapies in the treatment of ischemic heart disease.

The aim of this study was to determine the effect of tadalafil on miR‐21, cell survival and mobilization, and signaling pathways involved in tadalafil mediated effect on stem cell based repair of myocardial ischemia.

## Methods

This study was carried out in accordance with the recommendations in the Guide for the Care and Use of Laboratory Animals of the National Institutes of Health (NIH Publication *#*85‐230, revised 1996). The protocol was approved by the Institutional Animal Care and Use Committee (IACUC), University of Cincinnati (Protocol Number: 12‐04‐17‐01). All surgery was performed under anesthesia, and every effort was made to minimize suffering.

### In vitro study

BM‐derived MSCs were isolated from Fisher‐344 rats by flushing femur and tibia cavities, and were confirmed by higher expressions of CD44, CD90 and CD29 and lower c‐kit, CD31and CD45 as described previously (Haider et al. 2010a).

#### Cell viability assays

MSCs were seeded at 4 × 10^4^ cells in FB96‐well cell culture plates. Cells were deprived of serum and glucose for 12 h before exposure to the PDE5 inhibitor (tadalafil, *Cialis*™ Eli Lilly, USA). Firstly, the toxicity of tadalafil to MSCs (0–200 *μ*mol/L) and exposure durations (1–4 h) were evaluated by lactic dehydrogenase (LDH, cell necrosis) release assay (Promega), cell proliferation assays as Cell Counting Kit‐8 (CCK‐8 kit; Dojindo) and terminal transferase‐mediated dUTP‐X nick end labeling (TUNEL, In Situ Cell Death Detection Kit, TMR red; Roche), according to the manufacturer's instructions. The number of TUNEL‐positive (TUNEL^+^) nuclei was counted in at least five randomly selected high‐power fields (×200) with three independent samples. We found that tadalafil was non‐cytotoxic on MSCs up to 100 *μ*mol/L. For the current studies, we pre‐exposed MSCs to various pharmacological inhibitors followed by 1 *μ*mol/L tadalafil for 2 h prior to exposure to H_2_O_2_ (100 *μ*mol/L, an oxidative stress agent) for 4 h. The pharmacological inhibitors of PKG1 (KT5823, 1 *μ*mol/L; Sigma), MAPK (U0126, 25 *μ*mol/L; Calbiochem), NO synthase (NOS) (L‐NAME, 6 *μ*mol/L, Sigma‐Aldrich), CXCR4 (AMD3100, 5 *μ*g/mL; Sigma‐Aldrich), FasL (monoclonal anti‐FasL purified immunoglobulin, CD95L, 10 *μ*g/ml, Sigma), and miR‐21 with luciferase construct ± Fas (see below) were administered separately 2 h before tadalafil exposure. After 2 h of tadalafil exposure, culture media were replaced with 100 *μ*mol/L H_2_O_2_ diluted in serum‐ and glucose free‐ media for an additional 4 h at 37°C in a 5% CO_2_ and 95% N_2_ atmosphere. As controls, MSCs were exposed throughout the studies in randomized fashion to ± tadalafil (1 *μ*mol/L) for 2 h with or without DMSO or H_2_O_2_ (100 *μ*mol/L for 4 h).

#### PCR microarray and RT‐PCR miR‐21 assay

We used microarrays to identify relevant miRNAs implicated in cytoprotection after treatment with tadalafil. Total RNA, including miRNAs, was extracted using *mir*Vana miR isolation kit (Ambion). Samples, each containing 4 *μ*g RNA, were obtained from tadalafil‐treated MSCs (MSCs^T^) and nontreated control MSCs (MSCs^C^) and sent to LC Sciences (Houston, TX, USA) for miR microarray profiling. Data were analyzed by LC Sciences with proprietary computer programs. The expression of miR‐21/U6 was detected using *mir*Vana qRT‐PCR miRNA detection kit (Ambion). Specific miR‐21 and U6 primers from Ambion were used for RT‐PCR. The expression of U6 was used as endogenous control for each sample. Briefly, the reaction mixture (10 *μ*L) contained: 2 *μ*L of *mir*Vana 5 ×  RT buffer, 1 *μ*L of miR‐21 RT primer, 0.4 *μ*L of Array Script Enzyme Mix, 0.2 *μ*L of total RNA, and 6.4 *μ*L of H_2_O. Samples were incubated at 37°C for 30 min, followed by 95°C for 10 min. The program was set at 95°C for 30 min, followed by 40 cycles of 95°C for 15 sec, followed by 60°C for 30 sec. The bands were quantitatively assessed by densitometer.

#### MSCs transfection with the miR‐21 inhibitor

A precursor miR‐21 expression clone was constructed in a feline immunodeficiency virus‐based lentiviral vector system (pEZX‐MR04‐miR‐21) with a luciferase reporter construct containing the 3′‐UTR of Fas (TNF receptor super family member) designed to encompass the miR‐21 binding sites (pEZX‐Luc‐Fas 3′‐UTR, GeneCopoeia™, Rockville, MD) (Suzuki et al. 2010). MSCs were plated in triplicate into 24‐well plates and co‐transfected with 0.8 *μ*g of pEZX‐miR‐21 or pEZX‐miR‐ scrambled (Sc) control clone for pEZX‐AM04 (GeneCopoeia™). A luciferase reporter construct was created using Lipofectamine 2000™ (Invitrogen). Then, luciferase activities were measured in transfected MSCs treated ± tadalafil using the Dual Luciferase Reporter Assay System kit (Promega) and cell status assays (CCK‐8 and TUNEL), according to manufacturer's instructions. To knock down miR‐21, MSCs were transfected with anti‐miR‐21 (miRNA inhibitor; Ambion) and siPORT™ NeoFx™ transfection agent (NeoFx; Ambion) according to manufacturer's instructions (Haider et al. 2010b; Suzuki et al. 2010). Briefly, 5 *μ*L of siPORT™ NeoFx was diluted with 100 *μ*L of Opti‐MEM for 10 min. The anti‐miR inhibitor was then diluted with 100 *μ*L of Opti‐MEM to a final concentration of 30 nmol/L and added to the diluted NeoFx mixture. After another 10 min, the transfection mixture was added to 2.3 mL of cell suspension (3 × 10^5^ cells). Each cell suspension containing transfection mixture was plated into a FB6‐well plate of cells and incubated at 37°C with DMSO ± tadalafil for 48 h. Fas and STAT3 expression were assessed in MSCs extract.

### In vivo study

In this study, 150 young female Fischer rats (150–200 g) and 20 young C57BL6/J mice (25–30 g) were obtained from Harlan. Acute Myocardial infarction (AMI) was induced by the permanent ligation of the left anterior descending (LAD) coronary artery as described previously (Elmadbouh et al. 2007). The animal mortality rate after interventions (LAD ligation or myeloablation) was 20–35% and we were able to get stable numbers (*n* = 8 animal/group) in all experimental groups.

#### Cardioprotective effect of tadalafil on acute myocardial infarction (AMI) in vivo

AMI rats were assigned to 3 groups (*n* = 16/group) to receive multiple intramyocardial injections of 70 *μ*L DMEM alone (control) or with 1.5 x 10^6^ cells alone nontreated (MSCs^C^) or tadalafil treated MSCs (MSCs^T^) into infarcted and peri‐infarcted area. The whole LV tissue samples from each group (*n* = 8/group), harvested at day 7 for ELISA and western blots. Echocardiography was evaluated in rats at 2 weeks and 1 month (*n* = 8/group) (see below).

#### Mobilization and homing effect of tadalafil through NO/CXCR4 pathways

A) AMI rats were randomly assigned to four groups (*n* = 8/group) and were treated with IP injections (in volume 250 *μ*L) for 5 days as follow: saline (control), tadalafil (5 mg/kg/day; half‐life, 17.5 h), NO synthase inhibitor (L‐NAME, 150 *μ*g/kg) + tadalafil (5 mg/kg/day) or CXCR4 inhibitor (AMD3100, 5 mg/kg; half‐life, 3.5 h) + tadalafil (5 mg/kg/day). AMD3100 or L‐NAME treatment was started 2 h before tadalafil treatment. Echocardiography was evaluated in rats at 2 weeks and 1 month (*n* = 8/group) (see below).

B) Female rats were irradiated with cesium (600 cGy × 2 times, total 12000 cGy, Elite Gammacell 1000) for myeloablation. One day after irradiation, the recipient rats were intravenously (IV) injected with MSCs (5 × 10^6^) from donor male transgenic rats BM expressing green fluorescent protein (GFP) via tail vein for BM reconstitution as previously described (Zhao et al. 2009). One month post‐myeloablation, rats were subjected to LAD ligation and were randomly assigned to 4 groups (*n* = 8/group) as follows: ± 5 daily IP with tadalafil and/or preceded by L‐NAME and AMD3100, and then were euthanatized at day 7 for assessing ELISA and western blots of LV extracts (see below).

C) LAD ligation of female mice was performed. After 24 h, AMI mice were treated by IP injections of tadalafil (20 mg/kg in 250 *μ*L, *n* = 8) or saline (control *n* = 8) for 1 h; and then followed by IV injection of male donor mice MSCs expressing GFP, 2 × 10^6^ labeled with 37 MBq^111^In‐oxine (171 and 245 keV, ^111^In‐oxine half‐life is 2.8‐days; Nycomed Amersham) via the tail. Mice were used instead of rats due to the size pinhole collimator to obtain a sufficiently high spatial resolution CT/SPECT image in Cincinnati radiology department. In vitro, the labeling efficiency [LE = counts labeled cells/(counts labeled cells + counts supernatant) ×100] and cell viability (trypan blue test) was > 95.2% and 90%, respectively. For scintigraphic imaging, animals were anesthetized with intramuscular injection of ketamine (100 mg/kg, Curamed) and midazolam (2 mg/kg, Hoffmann LaRoche). Combined SPECT/CT imaging was performed for assessing fate of transplanted ^111^In‐oxine–labeled MSCs in response to ± tadalafil at 2 h, 24 h, and 48 h. All mice were euthanatized at day 7 post AMI, bio‐distribution assays of selected organs (hearts, lungs, kidneys, liver, and spleen) and the specific ^111^In‐oxine activity (megabecquerel per gram of tissue after correction for radioactive decay, MBq/g tissue) was performed in ± tadalafil‐treated mice. Also, the ex‐vivo fluorescent imaging for detection of grafted GFP cells was carried out in all hearts in both groups.

### Echocardiographic assessment

Transthoracic echocardiography was performed on rats transplanted with MSCs preconditioned with tadalafil or injected with IP tadalafil ± L‐NAME and AMD3100 after 2 weeks and 1 month. Each animal was anesthetized with pentobarbital (25 mg/kg, IP) and placed in supine position. Echocardiography was performed using compact linear array probe CL 10‐5 on HDI/5000 SONOS CT (HT‐Company) for parasternal long‐axis and short‐axis views, by an observer blinded to the treatment groups. Anterior and posterior end‐diastolic and end‐systolic wall thickness, LV end‐systolic and end‐diastolic diameters (LV‐ESD and LV‐EDD) and volumes (LV‐ESV and LV‐EDV) were measured from at least three consecutive cardiac cycles. Indices of LV systolic functions including fractional shortening (LV‐FS%) and ejection fractions (LV‐EF%) were calculated using LV‐FS = (LV‐EDD‐LV‐ESD)/LV‐EDD ×100 and LV‐EF = [(LV‐EDD^3^‐LV‐EDD^3^)/LV‐EDD^3^] × 100, respectively.

### ELISA and western blot analysis

MSCs were collected using cell culture scraper in Pierce^®^ RIPA buffer (Thermo Fisher Scientific, USA) containing protease inhibitors (10 *μ*g/mL aprotinin, 1 mmol/L phenylmethylsulfonyl fluoride, 10 *μ*g/mL leupeptin), and phosphatase inhibitors (50 mmol/L sodium fluoride, 1 mmol/L sodium orthovanadate, 10 mmol/L sodium pyrophosphate). For in vivo studies, the whole LV tissue samples from each group, harvested at day 7, were homogenized in 0.1 mol/L Tris, 4 mmol/L EDTA, 0.1% Triton X‐100, pH 7.6 buffer, sonicated and centrifuged (11000xg, 15 min at 4°C) to protein extracts as previously described (Elmadbouh et al. 2007). Protein was measured using Pierce™ BCA protein assay kit (Thermo Fisher Scientific, USA). The level of NO was assessed by NO assay (Abcam Inc, USA) that measure nitrate/nitrite concentration, NOS assay (Calbiochem), cGMP activity (Amersham) and hSDF‐1*α* (R&D systems) were performed on MSCs and LV tissue extracts according to the manufacturer's instructions, and the results were expressed per mg protein. Protein samples (40 *μ*g) were fractionated by 12% SDS‐polyacrylamide gel electrophoresis (ISC BioExpress) and electro‐transferred onto a nitrocellulose membrane. The membrane was blocked for 1 h with 1xTBS blocking buffer (Cell Signaling Technology) and 5% nonfat dry milk, followed by incubation with gentle shaking at 4°C with the primary antibodies diluted in blocking buffer. The primary antibodies used in western blot included: total Akt and phospho‐Akt (1:1000), phospho‐GSK3*β* (pGSK3*β* and GSK3*β*, 1:2000, Cell Signaling Technology); iNOS (1:1000, BD Biosciences), CXCR4 (1:300, Abcam), SDF‐1*α* (1:200, R&D Systems), and PKG1 (cGMP‐dependent, type 1, PRKG1, 1:200), Bcl2 (1:200), VEGF (1:500), extracellular signal‐regulated kinase 1/2 (phospho‐Erk1/2, phosphor‐P44/42 MAPK (thr 202/tyr 204) antibody, 1:1000), phospho‐VASP, signal transducers and activators of transcription‐3 (phospho‐STAT3), Bcl‐xl, Fas, and *α*‐actin (1:1000), (Santa Cruz Biotechnology). Actin served as the loading control. The membrane was washed 3 times for 5 min each with 1xTBS blocking buffer and 0.1% Tween^®^ (TBS/T). The primary antibody reaction was detected by incubating for 1 h with horseradish peroxidase (HRP)‐conjugated secondary antibody (1:2000) and HRP‐conjugated anti‐biotin antibody (1:1000, Cell Signaling Technology) diluted in TBS/T with 5% nonfat dry milk. The membrane was washed and developed using SuperSignal™ West Pico Plus chemiluminescent substrate (Thermo Fisher Scientific, USA). The chemiluminescent signal from blots has been detected by FluorChem™ E System (Thermo Fisher Scientific, USA), and were quantitatively assessed by densitometer.

### Statistical analysis

All experiments were performed at least 3 times, and all variable data were expressed as mean ± SEM using IBM SPSS^®^ Statistic version 20. Student's *t*‐test was used for comparison of unpaired data between tadalafil and control groups. One‐way analysis of variance (ANOVA) was used to compare unpaired data between 3 or more groups; followed by Bonferroni post hoc test for comparisons between corresponding time points in the two groups. Discrete variables were expressed as percentage and the Chi‐square or Fisher's exact test was used accordingly. A *P *< 0.05 was considered statistically significant.

## Results

### Preconditioning with tadalafil enhances cytoprotection of MSCs in vitro

**Figure 1:**
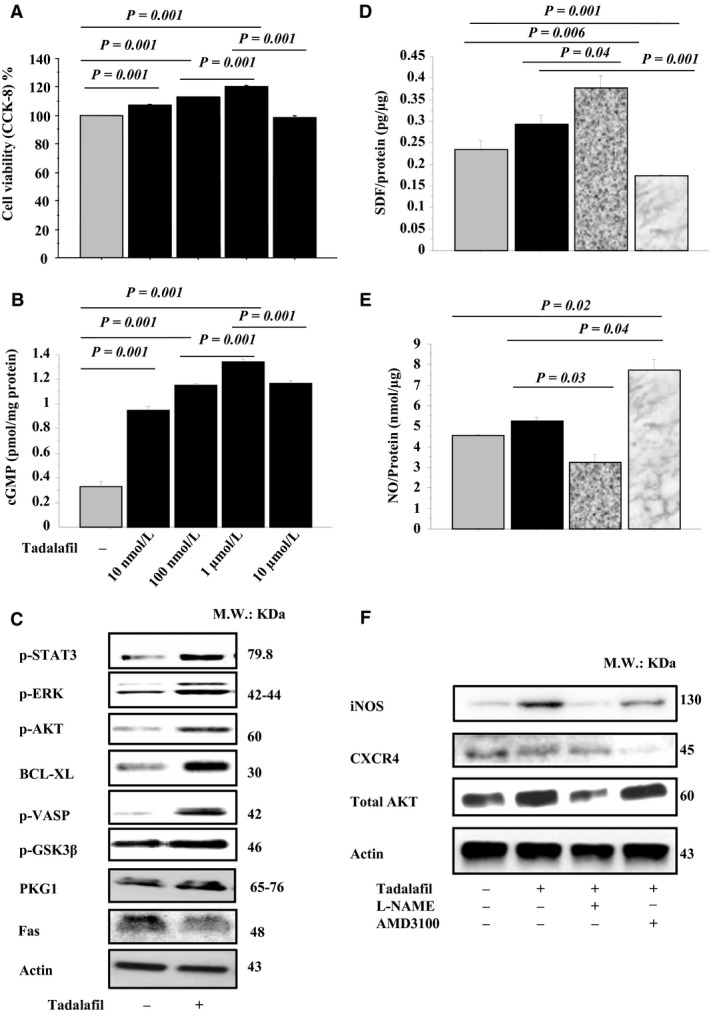
Effect of tadalafil treatment on in vitro MSCs: MSCs viability (CCK‐8 Assay, A) and cGMP activity (B) were assessed. The expression of p‐STAT3, p‐Erk1/2, p‐Akt, Bcl‐xl, p‐VASP, p‐GSK *β*, PKG1, and Fas (C) were assessed in MSCs extracts by western blots. Also, in MSCs extracts, SDF‐1*α* (D) and NO levels (E) (ELISA assay), and iNOS, CXCR4 and total‐Akt expressions (western blots assay) were assessed in ± NOS (L‐NAME) or CXCR4 (AMD3100) inhibitors (F). Values are mean ± SE;* n* = 6; *P‐*value <0.05 is significant.

In vitro cell cultures viability was enhanced by tadalafil in MSCs^T^ versus MSCs^C^ (Fig. 1A). cGMP activity was increased in MSCs treated by tadalafil in a dose‐dependent manner (Fig. 1B). Cell signaling (p‐VASP, Erk1/2, PKG1, p‐GSK3*β,* and p‐STAT3), and anti‐apoptotic molecules (higher of p‐Akt and Bcl‐xl; lower Fas expression) were enhanced by tadalafil in MSCs^T^ versus MSCs^C^ (Fig. 1C; Fig S1A). The concentration of SDF‐1*α*, NO, iNOS, Akt, CXCR4 was higher in MSCs^T^ versus MSCs^C^. In MSCs^T^ pretreated with L‐NAME, level of NO, total‐Akt, and CXCR4 was reduced, while SDF‐1*α* was unexpectedly higher. Also, AMD3100 pretreated MSCs^T^ had lower CXCR4 and SDF‐1*α* expression, but unexpectedly higher level of iNOS, NO, and total‐Akt were observed (Fig. 1D–F; Fig. S1BJ‐L).

**Figure 2:**
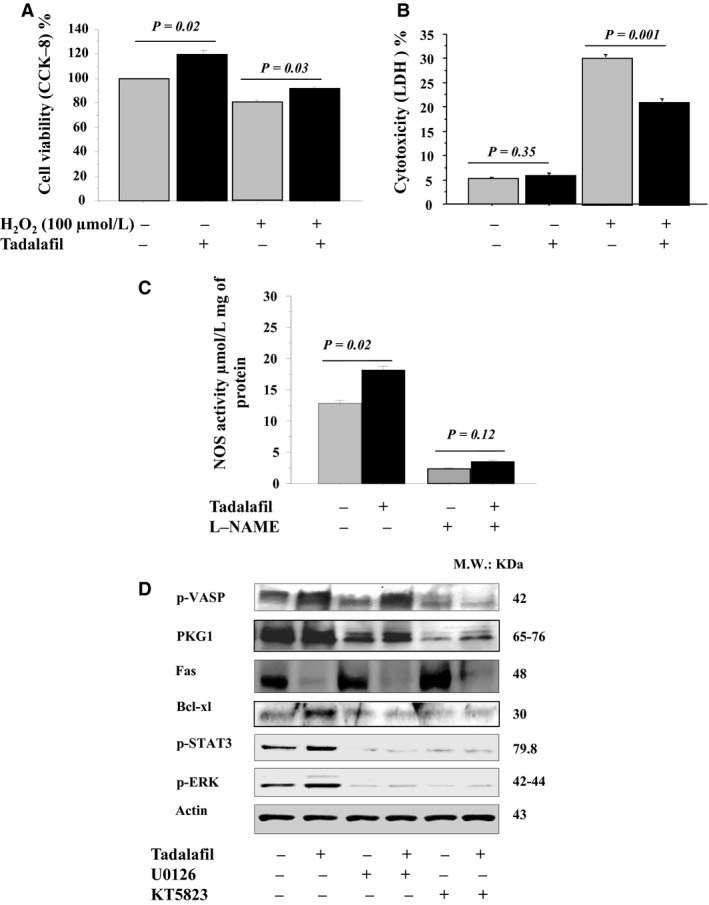
Tadalafil induced cytoprotection of in vitro MSCs under oxidative stress through PKG‐MAPK signaling pathways: Cell viability (CCK‐8 assay) (A) and cell death (LDH assay) (B) of MSCs after exposure to 100 *μ*mol/L H_2_O_2_ for 4 h (oxidative stress) were assessed in control and tadalafil. The level of NOS (ELISA assay, C) levels were assessed under oxidative stress in control and tadalafil in ± L‐NAME inhibitors. p‐VASP, PKG1, Fas, BcL‐xl, p‐ p‐STAT3 and Erk1/2 expressions (western blots, D) were assessed under oxidative stress in control and tadalafil in ± MAPK (U0126) and PKG1 (KT5823) inhibitors. Values are mean ± SE;* n* = 6; *P‐*value < 0.05 is significant.

Higher cell survival (Fig. 2A) and lower cell death (Fig. 2B) were observed in vitro MSC^T^ cultures exposed to oxidative stress (100 *μ*mol/L H_2_O_2_). MSCs^T^ had higher NOS levels when compared with MSCs^C^, but after treatment with L‐NAME inhibitors, the expression was abolished (Fig. 2C). Fas regulation by tadalafil was directly modulated by PKG‐MAPK signaling pathway. MSCs^T^ had higher p‐VASP, PKG1, Bcl‐xl, p‐Erk1/2 (Thr202/Tyr204) and p‐STAT3 (Tyr705) expression, but lower Fas expression versus MSCs^C^ (Fig. 2D; Fig. S2). However, these cytoprotective effects of tadalafil disappeared in MSCs^T^ after treatment with MAPK or PKG inhibitors when compared with MSCs^C^ (Fig. 2C–D). Also, Fas expression was reduced in MSCs^T^, and was restored by KT5823 and U0126 inhibitors (Fig. 2D; Fig. S2).

**Figure 3:**
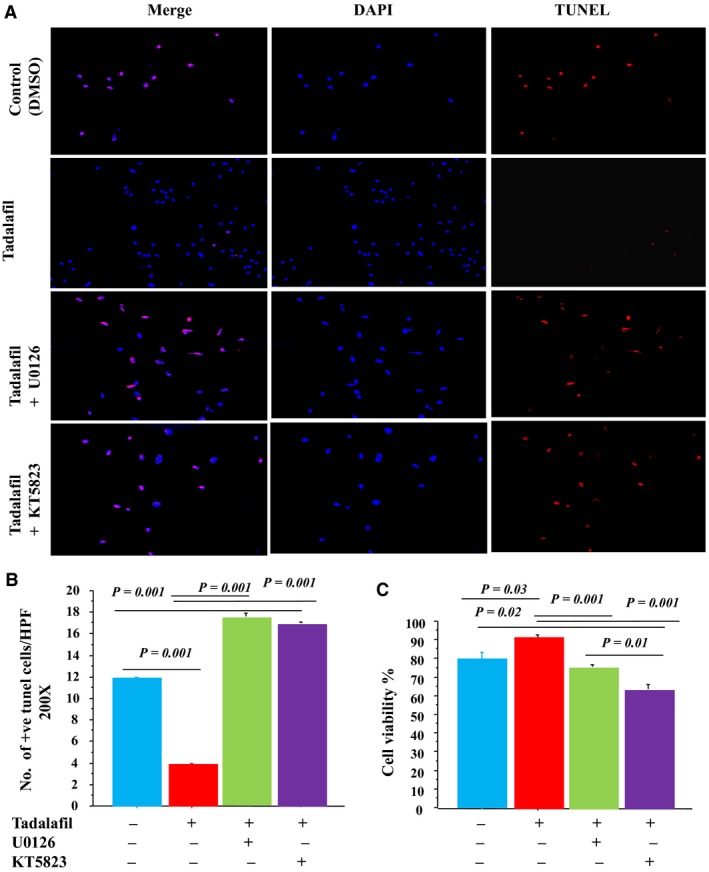
Tadalafil induced cytoprotection of MSCs under oxidative stress through an anti‐apoptotic signaling pathway. Number of TUNEL ^+^ cells (A; ×200, Red = TUNEL ^+^ nuclei; blue=DAPI, B; TUNEL ^+^ cells numbers) and MSCs viability (CCK‐8 assay, C) were assessed in tadalafil‐treated versus DMSO‐treated MSCs in vitro after 4 h exposure to 100 *μ*mol/L H_2_O_2_ in ± MAPK (U0126) and PKG1 (KT5823) inhibitors. Values are mean ± SE;* n* = 6; *P‐*value < 0.05 is significant.

The number of apoptotic cells (TUNEL^+^ cells) was markedly reduced in MSCs^T^ versus MSCs^C^ (Fig. 3A–B). However, these cytoprotective effects of tadalafil were abolished by MAPK or PKG inhibitors (Fig. 3A–B). Cell survival was significantly increased in MSCs^T^ versus MSCs^C^ (Fig. 3C). Similarly, MAPK and PKG inhibitors reduced cell survival in cells treated with tadalafil (Fig. 3C).

**Figure 4:**
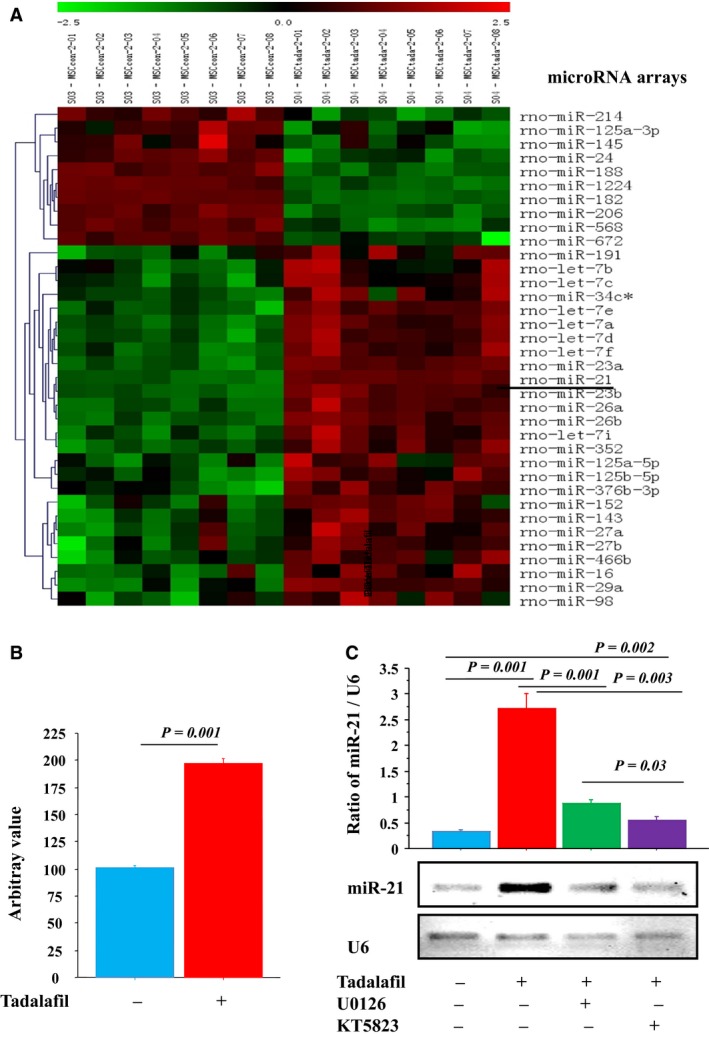
Tadalafil increased miR‐21 expression of in vitro MSCs under oxidative stress. Tadalafil induced miR‐21 up‐regulation (microRNA‐array analysis, (A). miR‐21 expression was increased in MSCs‐treated by tadalafil versus control (B). RT‐PCR: miR‐21 expression bands; and quantitative analysis of miR‐21/U6 expression was assessed in ± MAPK (U0126) and PKG1 (KT5823) inhibitors (PCR‐gel bands and densitometry assay, (C). Values are mean ± SE;* n* = 6; *P‐*value <0.05 is significant.

Among miR changes detected by microRNA array analysis, miR‐21 expression was upregulated in MSCs^T^ (Fig. 4A–B). The miR‐21/U6 band density was significantly increased in MSCs^T^ versus MSCs^C^, this upregulation was abrogated by PKG1 and MAPK inhibitors (KT5823 and U0126) (Fig. 4C).

**Figure 5:**
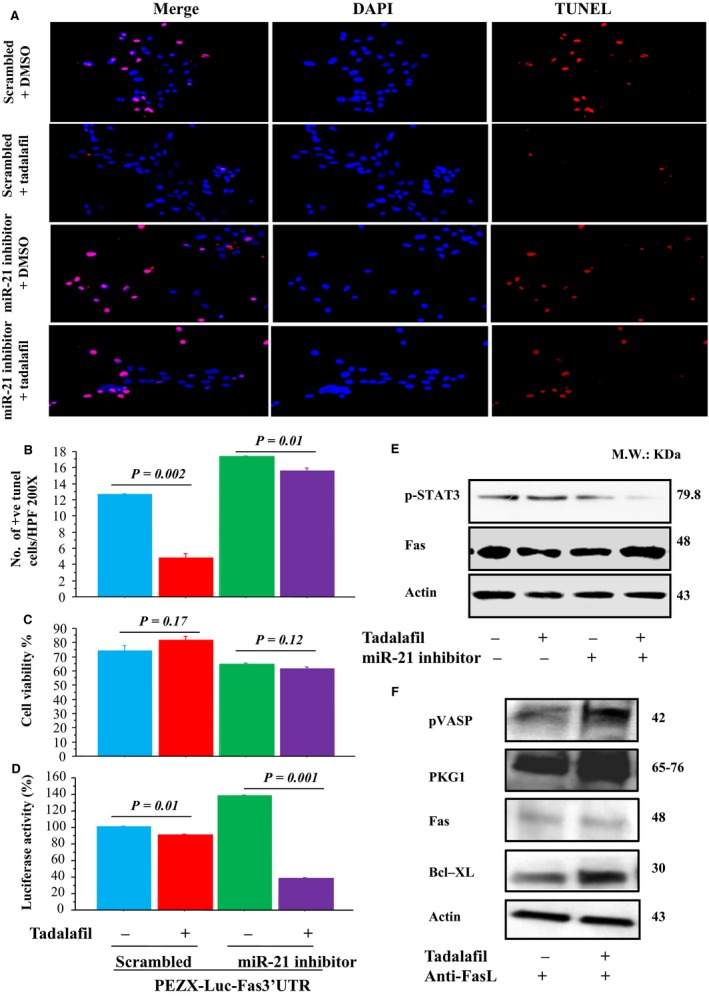
Cytoprotective effects of tadalafil were abrogated by miR‐21 inhibition in in vitro MSCs under oxidative stress. MSCs were transfected with pEZX‐Luc vector containing *Fas* 3′‐UTR together with a plasmid encoding miR‐21; or MSCs were transfected with anti‐miR‐21 using siPORT™ NeoFx™ transfection agent. Number of TUNEL ^+^ cells (A‐B) and cell viability (CCK‐8 assay, C), luciferase activity % (D), STAT3 and Fas expressions (western blots, E) were assessed after treatment with ± miR‐21 inhibitors. Also, p‐VASP, PKG1, Fas and BcL‐xl expressions (western blots, F) were assessed under oxidative stress in control and tadalafil in ± FasL inhibitors. Values are mean ± SE;* n* = 6; *P‐*value < 0.05 is significant.

Fewer apoptotic cells (TUNEL^+^ cells) and higher cell viability were observed in scrambled MSCs^T^, and was abrogated by miR‐21 inhibitors (Fig. 5A–C). Luciferase activity was significantly reduced in MSCs^T^ cotransfected with the plasmid containing the 3′‐UTR of the Fas gene (pEZX‐Luc‐Fas 3′‐UTR) with miR‐21 antisense versus MSCs^C^ ± non‐transfected with miR‐21 inhibitors (Fig. 5D). Also, after transfection of the MSCs with plasmid antisense miR‐21‐specific inhibitor using cationic vector, p‐STAT3 expression was increased in MSCs^T^ and was reduced by miR‐21 antisense. In contrast, Fas expression was reduced in MSCs^T^ and was recovered by miR‐21 antisense (Fig. 5E, Fig. S3A‐B). Also, Fas regulation by tadalafil was directly modulated by addition of FasL inhibitors in MSCs cultures. Lower Fas expression and higher p‐VASP, PKG1 and Bcl‐xl expressions in MSCs^T^ after treatment with FasL inhibitors when compared with MSCs^C^ (Fig. 5F; Fig. S3C‐F).

### Transplantation of MSCs^T^ improved grafted cell survival, LV remodeling and function in the infarcted hearts

**Figure 6:**
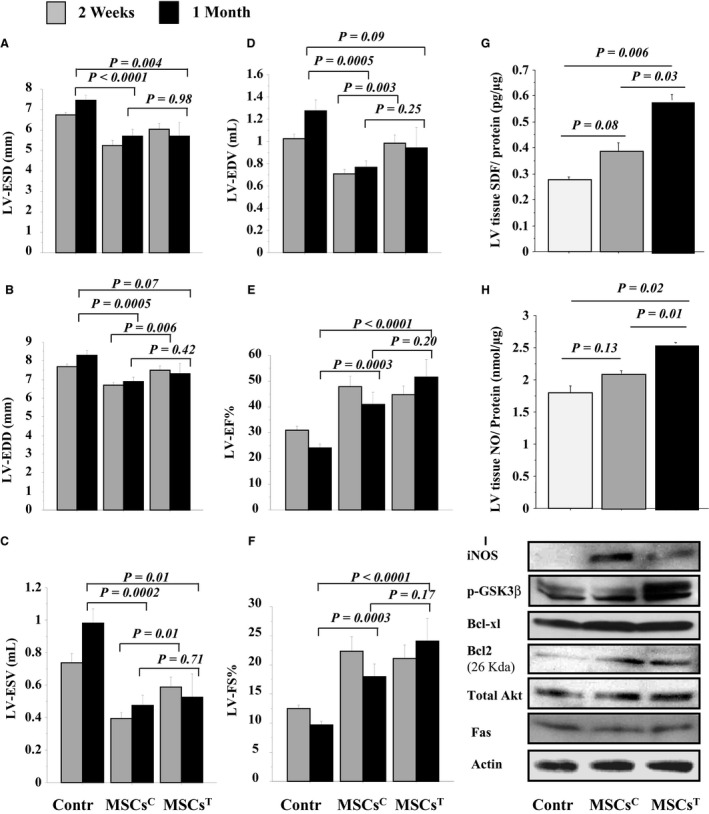
Tadalafil improved in vivo left ventricular (LV) function of infarcted hearts: LV‐ESD (A), LV‐EDD (B), LV‐ESV (C), LV‐EDV (D), LV‐EF (E), and LV‐FS (F) were assessed by echocardiography in control and MSCs transplanted ± tadalafil groups after 2 weeks and 1 month post‐treatment. At 7 days, both SDF‐1*α* (G) and NO (H) levels (ELISA assay), iNOS, p‐GSK *β*, Bcl‐xl, Bcl‐2, total‐Akt, and Fas expressions (western blots, I) were assessed in LV tissues extracts in control and MSCs transplanted ± tadalafil groups. Values are mean ± SE;* n* = 8; *P‐*value <0.05 is significant.

After 2 weeks, cardiac remodeling parameters (LV‐ESV and LV‐EDV; *P *=* *0.01, *P *=* *0.03, respectively) were significantly higher in MSCs^T^ ‐transplanted rats versus MSCs^C^ ‐transplanted. Moreover, after 1 month, cardiac remodeling parameters (LV‐EDD, LV‐ESD, LV‐ESV and LV‐EDV) were significantly improved in MSCs^T^ ‐transplanted rats versus DMEM control rats (*P *<* *0.05), higher nonsignificantly than MSCs^C^ ‐transplanted rats (*P *>* *0.5), thus indicating better preservation of LV contractile function (Fig. 6A–D). However, one‐way ANOVA analysis showed there was significant improvement in cardiac remodeling parameters in LV‐ESV and LV‐EDV (*P = 0.02, P = 0.001*, respectively) in MSCs^T^ ‐transplanted rats versus MSCs^C^ ‐transplanted rats. Also, heart function parameters (LV‐EF% and LV‐FS%) were significantly improved in MSCs^T^ ‐transplanted versus DMEM control (*P *<* *0.001) and higher nonsignificantly than in MSCs^C^ ‐transplanted rats (*P *>* *0.5) (Fig. 6E–F). However, one‐way ANOVA analysis showed there were significant differences between 2 weeks and 1 month in LV‐EDV (*P* = 0.04), LV‐ESV (*P* = 0.008) and LV‐EF% (*P* = 0.04). At day 7 post‐transplantation, level of SDF‐1*α* and NO (Fig. 6G–H), and the expression of iNOS, total‐Akt, Bcl2, Bcl‐xl, and p‐GSK3*β* were more significantly higher; while Fas expression was less (Fig. 6I; Fig. S4) in the infarcted hearts with MSCs^T^ (*P *<* *0*.01)* versus hearts with control saline‐treatments. Bcl‐xl and p‐GSK3*β* (*P *=* *0.0003, *P *=* *0.0001, respectively) was significantly higher in the infarcted hearts treated with MSCs^T^ versus hearts transplanted with MSCs^C^, but no‐significant difference in total‐Akt and Bcl2 (*P *>* *0*.05*). The iNOS activity was mildly expressed in MSCs^T^ ‐transplanted *hearts* versus MSCs^C^ ‐transplanted (*P *=* *0.0005) hearts but was highly expressed in treated hearts than control rats (*P *=* *0.0004) (Fig. 6I; Fig. S4).

### Tadalafil improved heart function, mobilization, and homing of MSCs in the infarcted heart

**Figure 7:**
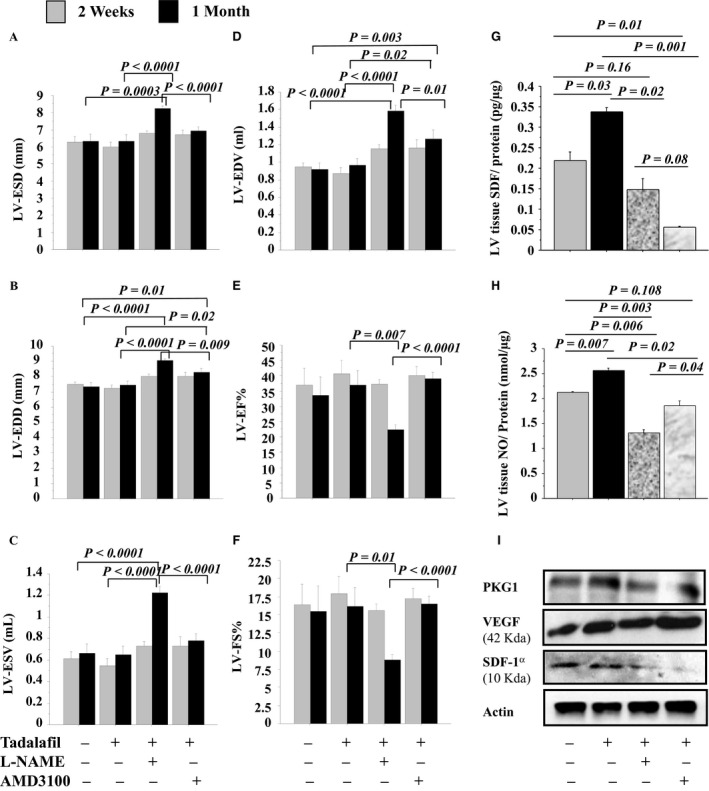
Tadalafil improved grafted cell survival in vivo and increased cell homing to the infarcted heart. LV‐ESD (A), LV‐EDD (B), LV‐ESV (C), LV‐EDV (D), LV‐EF% (E) and LV‐FS% (F) were assessed in control (Saline, IP) and IP tadalafil ± NOS (L‐NAME) or CXCR4 (AMD3100) inhibitors groups after 2 weeks and 1 month post‐treatment. In other groups, at 7 days, tadalafil effects on NO and CXCR4 signaling pathways were assessed after LAD ligation in 1 month post‐myeloablated rats with successful IV MSCs‐GFP ^+^. In the LV tissue extracts, SDF‐1*α* (G) and NO (H) levels (ELISA assay); and PKG1, VEGF and SDF‐1*α* expressions (western blots, I) were assessed in ± L‐NAME and AMD3100. Values are mean ± SE;* n* = 8; *P‐*value < 0.05 is significant.

Firstly, after 2 weeks and 1 month, the preservation of LV remodeling and function was observed in rats treated by IP tadalafil than in controls. However, the cardioprotective effects of tadalafil on heart function were abolished by L‐NAME or AMD3100 (Fig. 7A–F). However, one‐way ANOVA analysis showed a significant decrease in cardiac remodeling parameters in LV‐ESD (*P = 0.03*) in rats treated with AMD3100 than tadalafil IP. Also, there were significant differences in LV‐ESV (*P* = 0.005) and LV‐EF% between 2 weeks and 1 month (*P* = 0.04).

Secondly, at day 7, NO and SDF‐1*α*/CXCR4 signaling pathways were verified in AMI‐myeloablated rats after 5 days treatment with tadalafil ± L‐NAME or AMD3100. SDF‐1*α* and NO levels (Fig. 7G–H), and SDF‐1*α*, PKG1 and VEGF expressions were higher in LV tissue extracts of rats treated by IP tadalafil than in controls. In tadalafil treated rats: SDF‐1*α*, NO, and PKG1 were reduced by treatment with L‐NAME or AMD3100 except VEGF expression was increased with AMD3100, but decreased with L‐NAME (Fig. 7I; Fig. S5). Therefore, tadalafil improved recovery of ischemic myocardium through NO and SDF‐1*α*/CXCR4 signaling pathways important in homing of circulating stem cell/progenitors to the ischemic heart (Fig. S6).

**Figure 8:**
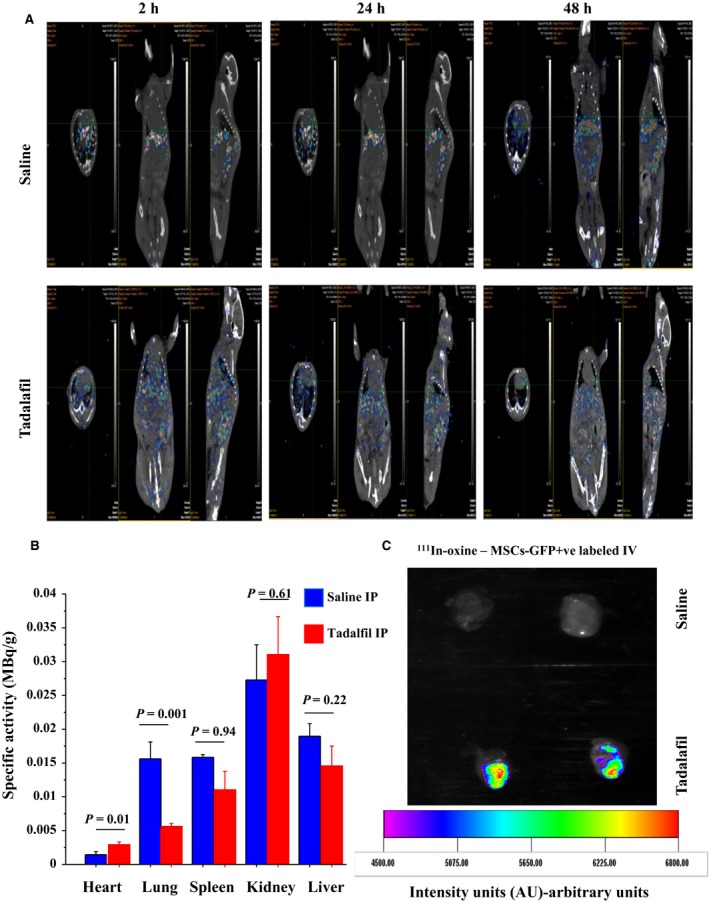
Tadalafil induced in vivo mobilization and homing of transplanted ^111^In‐oxine–labeled MSCs into infarcted heart. The SPECT/CT images of the chest and abdomin of mice were performed at 2 h, 24 h and 48 h to detect the uptake of ^111^In‐oxine–labeled MSCs‐GFP ^+^ in ischemia‐induced infarcted heart of mice injected intravenously (IV) 1 h after ± IP tadalafil (A). At day 7, the specific activity of ^111^In‐oxine was assessed in heart, lung, spleen, kidney and liver organs in tadalafil‐treated versus control mice (B). Ex‐vivo fluorescent imaging was assessed to detect the uptake of ^111^In‐oxine–labeled MSCs‐GFP ^+^ in hearts of mice treated with tadalafil versus controls (C). Values are mean ± SE;* n* = 8; *P‐*value < 0.05 is significant.

Thirdly, combined SPECT/CT imaging was performed at 2 h, 24 h, and 48 h for assessing fate of transplanted ^111^In‐oxine–labeled MSCs‐GFP^+^. A transient high lung uptake was observed within the first 2 h after infusion of MSCs in both groups. At 24 h after injection, the initial lung activity shifted toward heart, liver, kidneys, and spleen in the tadalafil versus controls (Fig. 8A). At day 7, the specific activity (MBq/g tissue) of heart increased in tadalafil (0.003 ± 0.001) versus control (0.001 ± 0.001) (*P *=* *0.01) in infarcted hearts. However, the overall specific radioactivity detected in the heart was with tadalafil treatment (17.5%) versus control (3.6%). The specific activity observed in the remaining organs with tadalafil treatment versus control was: lung (0.006 ± 0.001 *vs*. 0.016 ± 0.007, *P *=* *0.001), kidney (0.031 ± 0.015 *vs*. 0.027 ± 0.015, *P *=* *0.61), liver (0.015 ± 0.008 *vs*. 0.019 ± 0.005, *P *=* *0.22), and spleen (0.011 ± 0.007 *vs*. 0.016 ± 0.001, *P *=* *0.94) (Fig. 8B). *Ex vivo* fluorescent imaging revealed significant uptake of ^111^In‐oxine–labeled MSCs‐GFP^+^ in the tadalafil versus the control hearts (Fig. 8C). This result shows that the SPECT image of ^111^In‐oxine‐MSCs recruited or retained in the heart was co‐localized with grafted GFP^+^ cells in the same heart.

## Discussion

The current transplantation strategies achieve only minimal durable engraftment of donor stem cells in the infarcted myocardium, primarily due to the rather short life span of donor stem cells (Haider et al. 2008b; Elmadbouh et al. 2011).

In this study, the cytoprotective effect of tadalafil treatment increased MSCs survival with higher level of total‐Akt, p‐Akt, Bcl‐xl, Bcl2, cGMP, p‐Erk1/2, p‐VASP, p‐GSK3*β*, PKG1, NO, SDF‐1*α*, iNOS, and p‐STAT3; and lower expression of Fas. This cytoprotective of MSCs in vitro was decreased by pretreatment with L‐NAME and variably by AMD3100. The significant increase in p‐Erk1/2 and p‐STAT3 activity with tadalafil treatment was abrogated by pretreatment with PKG1 or MAPK inhibitors. Alleviated Fas protein by tadalafil was significantly recovered by PKG1 and MAPK inhibitors in agreement with previous studies (Ahmad et al. 2009; Salloum et al. 2009; Haider et al. 2010a; Li et al. 2012; Kumar and Ashraf 2015). VASP downstream of PKG1 may be involved in the intracellular signaling pathways. Also, the activation of p‐Erk1/2 and p‐STAT3 was involved in preconditioning‐induced PKG1 expression, and for its downstream effect on Fas pathway as in agreement with previous studies (Haider et al. 2010b; Kumar and Ashraf 2015).

Our results demonstrated that Fas has a critical role in apoptosis and is considered as one of the target of miR‐21 through MAPK signaling pathway. Preconditioning of MSCs with tadalafil significantly enhanced their survival by upregulation of miR‐21 that was reduced in the presence of PKG1‐MAPK and miR‐21 inhibitors. Also, we observed that the miR‐21 expression with tadalafil promoted survival of MSCs by directly targeting 3′‐UTR of Fas. These results implicate PKG1/MAPK signaling pathway has a role in miR‐21 regulation. This conclusion was consistent with previous studies suggesting that miR‐21 plays a critical role in anti‐apoptosis and can negatively regulate the Fas gene by stimulating MAPK signaling (Thum et al. 2008; Haider et al. 2010b). STAT3 is also activated by phosphorylation at Tyr705 and the transcriptional activation seems to be regulated by phosphorylation of STAT3 at Ser727 via the MAPK pathway (Kumar and Ashraf 2015). These data agree with our previous study results in diazoxide preconditioned skeletal myoblasts where miR‐21 was identified as a key anti‐apoptotic regulator acting via interleukin‐11‐induced Erk1/2–STAT3 signaling (Haider et al. 2010b). In another study, Fas was identified as an important target gene of miR‐146a, and that such targeting was related to an observed NF‐kB mediated anti‐apoptotic effect on BM‐derived stem cells (Suzuki et al. 2010). This conclusion was also consistent with another study that identified a unique involvement of Akt by which it inhibited apoptosis through miR‐21‐dependent suppression of FasL (Sayed et al. 2010; Zhang et al. 2012). Consequently, miR‐21 appears to play an important role in tadalafil‐induced protection against ischemic injury and apoptosis of the heart via regulating protection‐ or injury‐related genes such as inhibiting FasL, phosphatase, and tensin homolog deleted on chromosome ten (PTEN), tropomyosin 1, programmed cell death 4 (PDCD4), TAp63 isoform of the p53 family, sprouty 1 and 2, heterogeneous nuclear ribonucleoprotein K and LRRFIP1 (NF‐kB inhibitor) (Thum et al. 2008; Sayed et al. 2010).

The results from the in vivo component of our study also confirm significant MSCs survival. When assessed at 1 month following the direct injection of tadalafil‐treated MSCs into rat infarcted myocardium, there was improved MSCs survival that was associated with an increased production of SDF‐1*α*, NO, p‐GSK3*β*, iNOS, and total‐Akt, Bcl2, Bcl‐xl and a decreased expression of Fas similar to previous studies (Ahmad et al. 2009; Salloum et al. 2009; Haider et al. 2010a; Li et al. 2012). The cardioprotection afforded by tadalafil may therefore be orchestrated through several different signaling pathways.

Recently, mobilization and recruitment of BMCs using growth factors (VEGF and SDF‐1*α*) or drugs (atorvastatin, G‐CSF, fucoidan or tadalafil) offers an alternative to the direct injection of stem cells (Manzo‐Silberman et al. 2011). The mobilization of progenitor cells from the BM and the peripheral homing process are coordinated by a complex set of mechanisms not yet completely identified. Certainly, the involvement of NO, cytokines, and chemokines has been documented and these signaling molecules appear to play a key role in myocardial regeneration in both animal models and humans (Moore et al. 2001). The role of tadalafil and stem cell homing in ischemic heart disease is incompletely understood. We investigated the homing effect of tadalafil‐mediated stem cell trafficking after AMI with a focus on the roles of NO and SDF‐1*α*/CXCR4 in myocardium. We observed an improvement in heart function after 5 daily IP tadalafil treatment or pretreatment with tadalafil and AMD3100. Heart functions (LV‐EF% and LV‐FS%) were reduced by L‐NAME pretreatment. It has been reported elsewhere that AMD3100 prolonged BM progenitor mobilization (CXCR4^+^ and Sca1^+^/Flk1^+^ cells) and improved recovery from ischemic/reperfusion (I/R) injury (Pasha et al. 2008). These benefits appeared to occur through combined effects of AMD3100 and upregulated BM VEGF‐eNOS expression likely through eNOS signaling (matrix metalloproteinase‐9 and soluble Kit ligand), which favors extracellular matrix proteolysis, and the consequent release of progenitor cells from the BM niches to systemic circulation. Our results were consistent with those reported in other studies that showed PDE‐5 inhibitors could induce a powerful protective effect against cardiac diseases including I/R‐injury (Sesti et al. 2007), AMI‐induced heart failure (Salloum et al. 2009) and doxorubicin‐induced cardiomyopathy (Prysyazhna et al. 2016). These studies demonstrated that chronic tadalafil treatment activated NO‐induced silent mating type information regulation 2 homolog (SIRT1), a histone deacetylase that regulates PGC‐1*α* signaling and attenuates mitochondrial dysfunction in type 2 diabetic hearts (Koka et al. 2014). Recently, NO inhalation combined with tadalafil during myocardial I/R conferred superior protection against I/R‐injury in mice. The associated increase in cGMP‐signaling after the combined treatment suggests the importance of this pathway for beneficial long term structural and functional myocardium remodeling. Therefore, such combined therapies may represent promising strategies for translational research to improve the outcome of I/R‐injury in patients (Lux et al. 2016).

In myeloablated rats with AMI, SDF‐1*α*, NO, PKG1, and VEGF expression was significantly higher in hearts treated by tadalafil versus controls. Tadalafil may participate in BM progenitor mobilization and homing needed for the recovery of ischemic myocardium through the SDF‐1*α*/CXCR4, NO/cGMP, and PKG1/MAPK signaling cascades (Ii et al. 2005; Ahmad et al. 2009; Salloum et al. 2009; Haider et al. 2010a; Li et al. 2012). Taken together, tadalafil has myriad cardioprotective effects in the ischemic heart including inflammation reduction, platelets aggregation inhibition (Varma et al. 2012), bone marrow stem cells/progenitors mobilization and homing enhancement (Foresta et al. 2006, 2010); apoptosis and necrosis (cardioprotective effects) attenuation (Ahmad et al. 2009; Salloum et al. 2009; Haider et al. 2010a; Li et al. 2012).

Our study is the first to report the salutary effects of tadalafil on MSC mobilization and homing in AMI. After IV injection of ^111^In‐oxine labeled‐MSCs, most MSCs were initially entrapped within the lung capillaries during their first pass. Over the next 48 h, a significant proportion of the labeled MSCs escaped from the lung capillary system and migrated to the tadalafil‐treated infarcted hearts (17.5%) versus controls (3.6%). However, we cannot exclude the potential negative impact of ^111^In‐oxine on the function and viability of MSCs. The limited cell accumulation in the hearts after 96 h was consistent with results reported in another study where ^111^In leaked from labeled cells or the labeled cells phagocytosed by the reticuloendothelial cells could have occurred once the labeled MSCs were trapped in the lung capillaries (Brenner et al. 2004). Nevertheless, ^111^In‐oxine‐labeled donor stem cells have been used for follow‐up or for monitoring the fate of grafted cells into ischemic tissue after cell transplantation up to 10 days in animals (Brenner et al. 2004) and humans (Caveliers et al. 2007). ^111^In‐oxine‐ hematopoietic progenitor cells (intracoronary injection) were mobilized up to 2.3‐fold (the specific activity) in the infarcted heart (Brenner et al. 2004). Another, the engrafted ^99^Tc‐labeled fetal cardiomyocyte (IV injection) differentiated into all major cells of cardiovascular lineage and promoted the cardiac regeneration via paracrine effects of growth factors on cardiac remodeling (Garikipati et al. 2014). ^111^In‐oxine is safe and may be clinically used for labeling stem cells as an alternate to magnetic resonance dyes, traditional gene reporters (*β*‐gal or alkaline phosphatase) or fluorescent dyes (Elmadbouh et al. 2004; Garikipati et al. 2014).

In conclusion, tadalafil treatment increased ex vivo MSCs survival via up‐regulation of miR‐21‐dependent suppression of Fas through the PKG‐MAPK signaling pathway. Tadalafil also improved the grafted MSCs survival in vivo and promoted mobilization and homing of MSCs to the ischemic heart via NO/cGMP, PKG1/MAPK, and SDF‐1*α*/CXCR4 cascades (Fig. S6). Labeling stem cells with ^111^In‐oxine offers an effective method for sequential monitoring of cell trafficking, distribution, and assessment of the homing effects of putative therapeutic drugs.

## Conflict of Interest

The authors declare no conflicts of interest.

## Data Accessibility

## Supporting information




**Figure S1A.** Effect of tadalafil treatment on in vitro MSCs: The expression of p‐STAT3 (S1‐A), p‐Erk1/2 (S1‐B), p‐Akt (S1‐C), Bcl‐xl (S1‐D), p‐VASP (S1‐E), p‐GSKβ (S1‐F), PKG1 (S1‐G), and Fas (S1‐H) in MSCs extracts (western blots bands, Fig 1C) were assessed by the densitometry.

**Figure S1B.** Effect of tadalafil treatment on in vitro MSCs: MSCs viability (CCK‐8 Assay, I) were assessed ± tadalafil with NOS (L‐NAME) or CXCR4 (AMD3100) inhibitors.

**Figure S2.** Tadalafil induced cytoprotection of in vitro MSCs under oxidative stress through PKG‐MAPK signaling pathways: p‐VASP (S2‐A), PKG1 (S2‐B), Fas (S2‐C), BcL‐xl (S2‐D), p‐STAT3 (S2‐E), and p‐Erk1/2 (S2‐F) expressions (western blots bands, Fig 2D) were assessed by densitometry under oxidative stress in control and tadalafil in ± MAPK (U0126) and PKG1 (KT5823) inhibitors.

**Figure S3.** Cytoprotective effects of tadalafil were abrogated by miR‐21 inhibition in in vitro MSCs under oxidative stress.

**Figure S4.** Tadalafil improved in vivo left ventricular (LV) function of infarcted hearts: At 7 days, iNOS (S4‐A), p‐GSKβ (S4‐B), Bcl‐xl (S4‐C), Bcl‐2 (S4‐D), total‐Akt (S4‐E), and Fas (S4‐F) expressions (western blots bands, Figure 6I) were assessed by densitometry in LV tissues extracts in control and MSCs transplanted ± tadalafil groups.

**Figure S5.** Tadalafil improved grafted cell survival in vivo and increased cell homing to the infarcted heart. In other groups, at 7 days, tadalafil effects on NO and CXCR4 signaling pathways were assessed after LAD ligation in 1 month post‐myeloablated rats with successful IV MSCs‐GFP^+^.

**Figure S6.** Proposed signaling pathways for tadalafil effect on stem cells transplantations in the infarcted myocardium.
