# Supplementary material for: Tadalafil, a long acting phosphodiesterase inhibitor, promotes bone marrow stem cell survival and their homing into ischemic myocardium for cardiac repair
Source: Physiol Rep. 2017 Nov 15;5(21):e13480. doi: 10.14814/phy2.13480 (PMC5688776; doi:10.14814/phy2.13480)
Supplement: Supplementary file 1 — Figure S1A. Effect of tadalafil treatment on in vitro MSCs: The expression of p‐STAT3 (S1‐A), p‐Erk1/2 (S1‐B), p‐Akt (S1‐C), Bcl‐xl (S1‐D), p‐VASP (S1‐E), p‐GSKβ (S1‐F), PKG1 (S1‐G), and Fas (S1‐H) in MSCs extracts (western blots bands, Fig 1C) were assessed by the densitometry. [file PHY2-5-e13480-s001.pptx]

## Slide 1
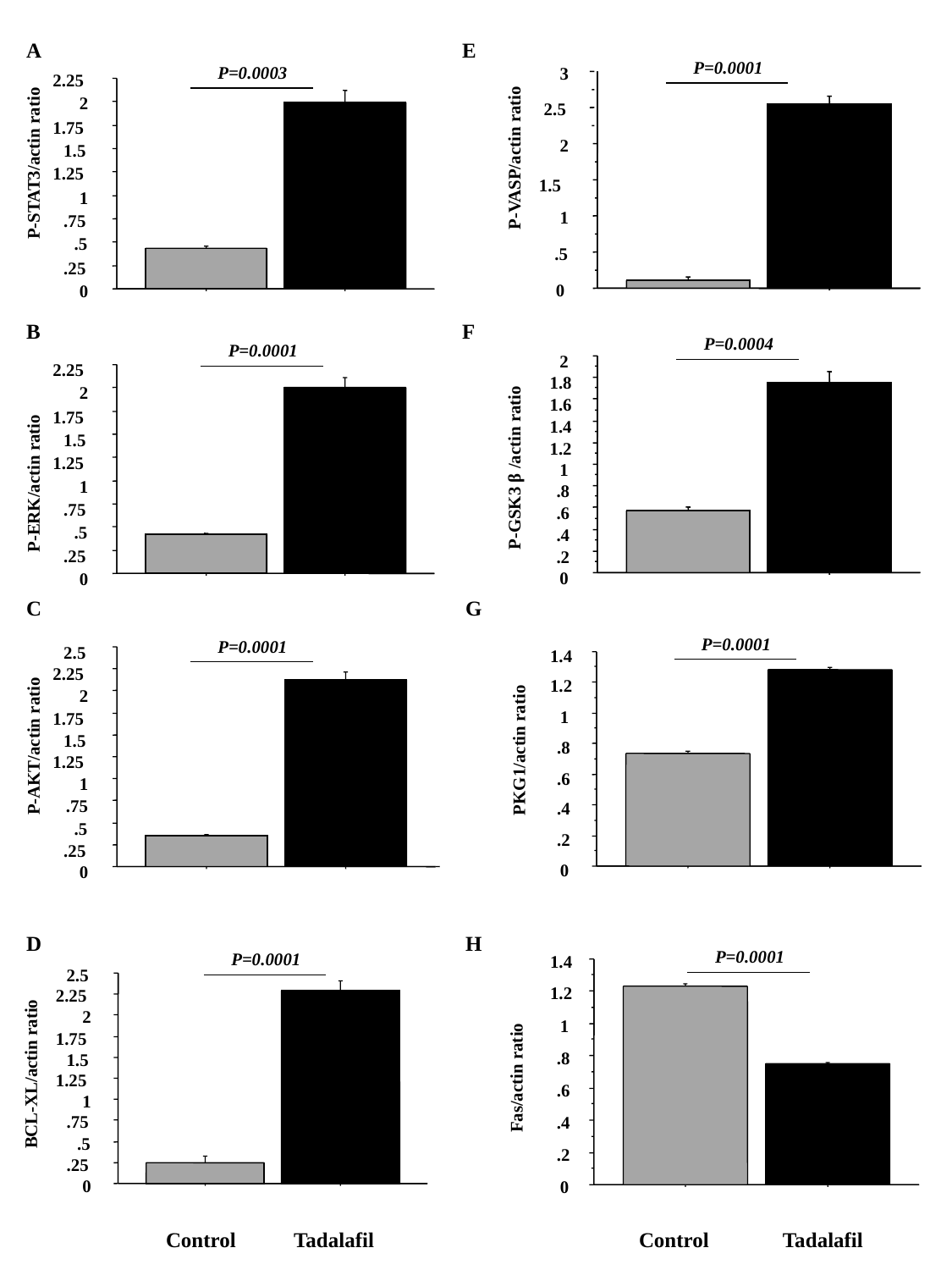

A
E
P=0.0001
P=0.0003
3
2.25
2
2.5
1.75
2
1.5
 P-VASP/actin ratio
P-STAT3/actin ratio
1.25
1.5
1
1
.75
.5
.5
.25
0
0
B
F
P=0.0004
2
1.8
1.6
1.4
1.2
P-GSK3 β /actin ratio
1
.8
.6
.4
.2
0
P=0.0001
2.25
2
1.75
1.5
1.25
P-ERK/actin ratio
1
.75
.5
.25
0
C
G
P=0.0001
P=0.0001
2.5
1.4
2.25
1.2
2
1
1.75
1.5
P-AKT/actin ratio
.8
PKG1/actin ratio
1.25
.6
1
.75
.4
.5
.2
.25
0
0
D
H
P=0.0001
P=0.0001
1.4
2.5
1.2
2.25
2
1
1.75
.8
1.5
BCL-XL/actin ratio
Fas/actin ratio
1.25
.6
1
.75
.4
.5
.2
.25
0
0
Control Tadalafil Control Tadalafil
