# Supplementary material for: Tadalafil, a long acting phosphodiesterase inhibitor, promotes bone marrow stem cell survival and their homing into ischemic myocardium for cardiac repair
Source: Physiol Rep. 2017 Nov 15;5(21):e13480. doi: 10.14814/phy2.13480 (PMC5688776; doi:10.14814/phy2.13480)
Supplement: Supplementary file 2 — Figure S1B. Effect of tadalafil treatment on in vitro MSCs: MSCs viability (CCK‐8 Assay, I) were assessed ± tadalafil with NOS (L‐NAME) or CXCR4 (AMD3100) inhibitors. [file PHY2-5-e13480-s002.pptx]

## Slide 1
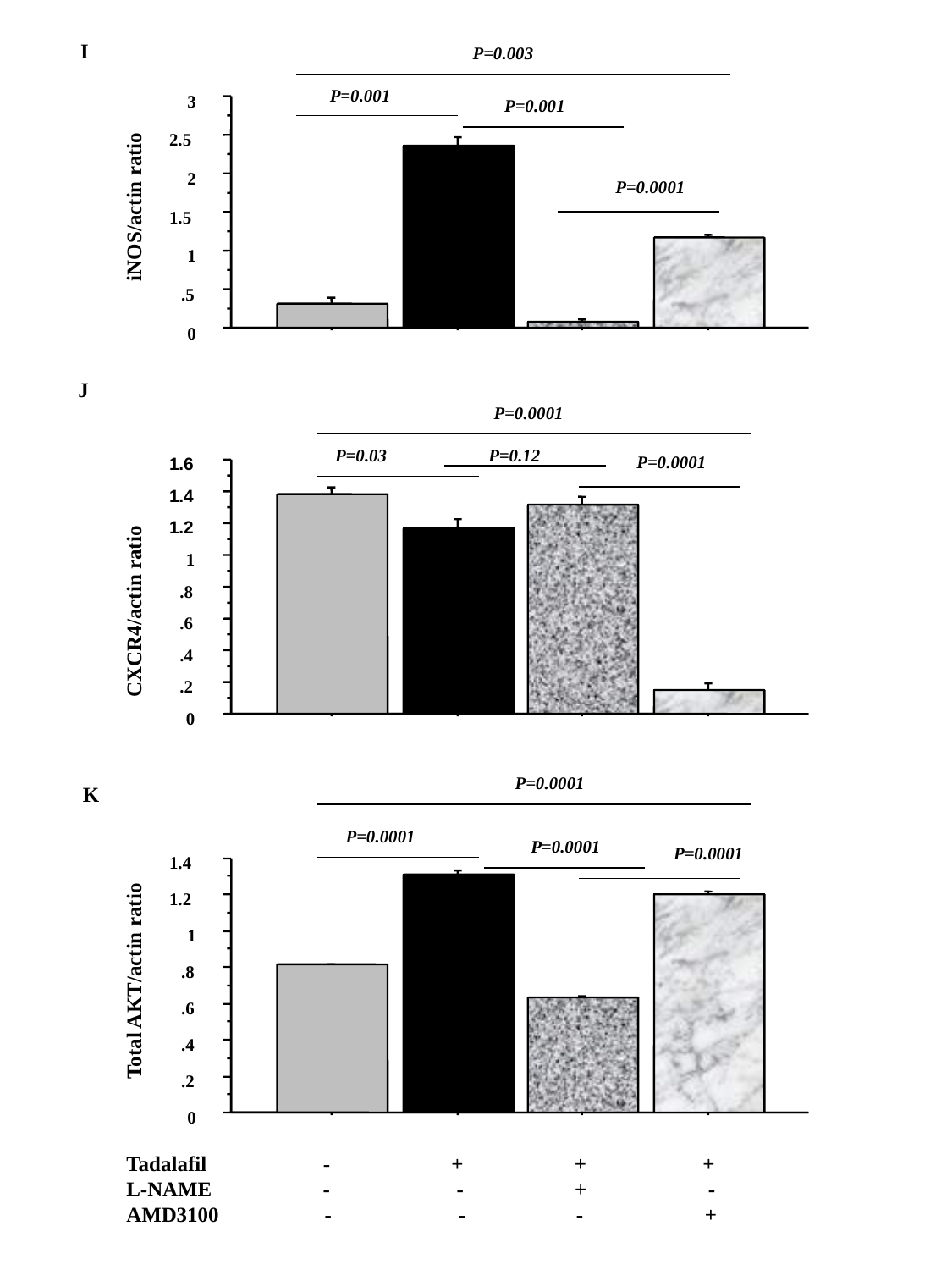

I
P=0.003
P=0.001
P=0.001
3
2.5
2
P=0.0001
iNOS/actin ratio
1.5
1
.5
0
J
P=0.0001
P=0.03
P=0.12
P=0.0001
1.6
1.4
1.2
1
.8
CXCR4/actin ratio
.6
.4
.2
0
P=0.0001
K
P=0.0001
P=0.0001
P=0.0001
1.4
1.2
1
.8
Total AKT/actin ratio
.6
.4
.2
0
Tadalafil - + + +
L-NAME - - + -
AMD3100 - - - +
