# Supplementary material for: Tadalafil, a long acting phosphodiesterase inhibitor, promotes bone marrow stem cell survival and their homing into ischemic myocardium for cardiac repair
Source: Physiol Rep. 2017 Nov 15;5(21):e13480. doi: 10.14814/phy2.13480 (PMC5688776; doi:10.14814/phy2.13480)
Supplement: Supplementary file 3 — Figure S2. Tadalafil induced cytoprotection of in vitro MSCs under oxidative stress through PKG‐MAPK signaling pathways: p‐VASP (S2‐A), PKG1 (S2‐B), Fas (S2‐C), BcL‐xl (S2‐D), p‐STAT3 (S2‐E), and p‐Erk1/2 (S2‐F) expressions (western blots bands, Fig 2D) were assessed by densitometry under oxidative stress in control and tadalafil in ± MAPK (U0126) and PKG1 (KT5823) inhibitors. [file PHY2-5-e13480-s003.pptx]

## Slide 1
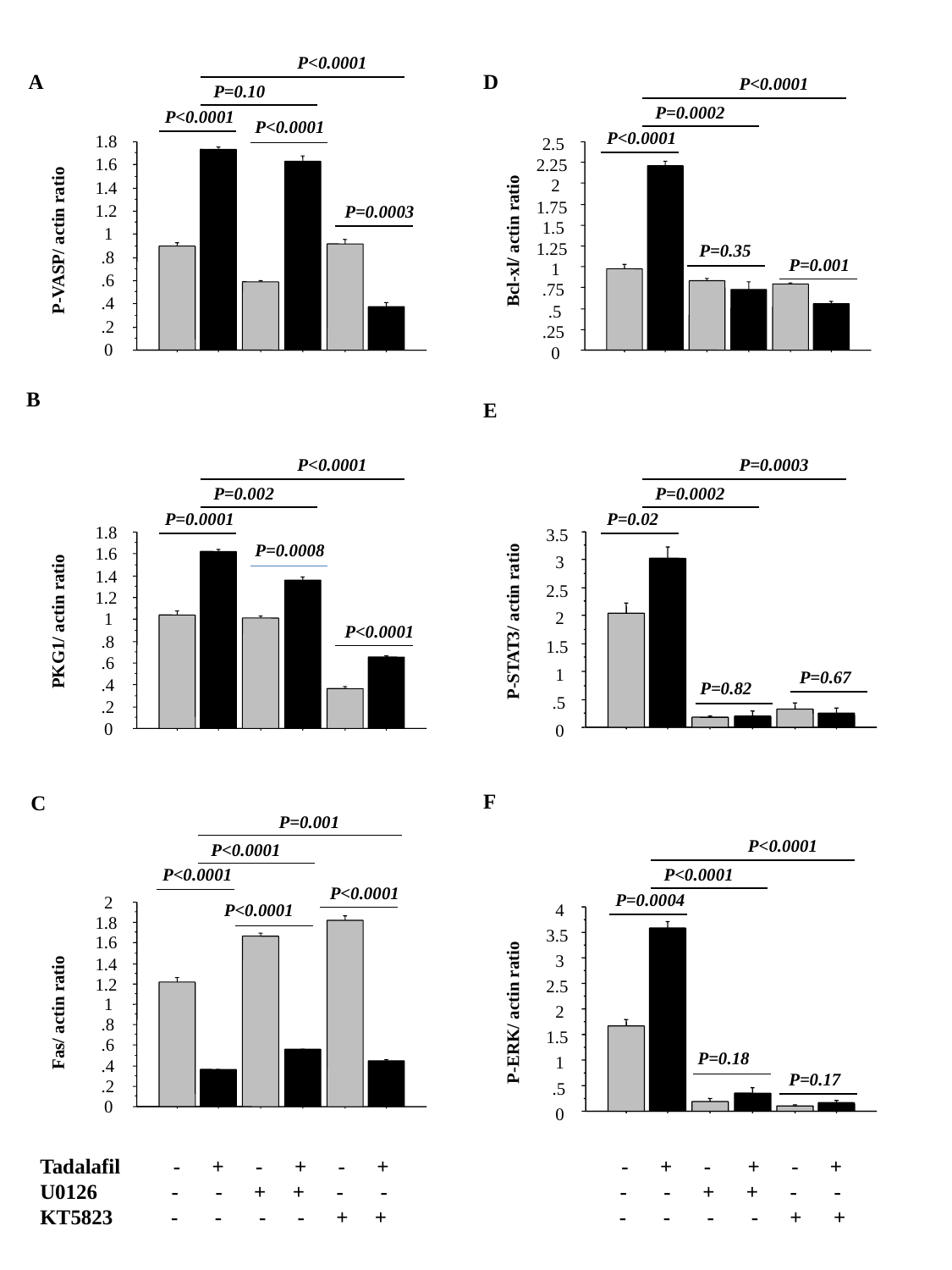

P<0.0001
A
D
P<0.0001
P=0.10
P=0.0002
P<0.0001
P<0.0001
P<0.0001
1.8
2.5
1.6
2.25
2
1.4
P=0.0003
1.75
1.2
1.5
1
Bcl-xl/ actin ratio
P-VASP/ actin ratio
P=0.35
1.25
.8
P=0.001
1
.6
.75
.4
.5
.2
.25
0
0
B
E
P<0.0001
P=0.0003
P=0.002
P=0.0002
P=0.0001
P=0.02
1.8
3.5
P=0.0008
1.6
3
1.4
2.5
1.2
2
1
P-STAT3/ actin ratio
PKG1/ actin ratio
P<0.0001
.8
1.5
.6
P=0.67
1
P=0.82
.4
.5
.2
0
0
F
C
P=0.001
P<0.0001
P<0.0001
P<0.0001
P<0.0001
P<0.0001
P=0.0004
2
P<0.0001
4
1.8
3.5
1.6
3
1.4
1.2
2.5
1
P-ERK/ actin ratio
Fas/ actin ratio
2
.8
1.5
.6
P=0.18
1
.4
P=0.17
.2
.5
0
0
Tadalafil - + - + - + - + - + - +
U0126 - - + + - - - - + + - -
KT5823 - - - - + + - - - - + +
