# Supplementary material for: Tadalafil, a long acting phosphodiesterase inhibitor, promotes bone marrow stem cell survival and their homing into ischemic myocardium for cardiac repair
Source: Physiol Rep. 2017 Nov 15;5(21):e13480. doi: 10.14814/phy2.13480 (PMC5688776; doi:10.14814/phy2.13480)
Supplement: Supplementary file 4 — Figure S3. Cytoprotective effects of tadalafil were abrogated by miR‐21 inhibition in in vitro MSCs under oxidative stress. [file PHY2-5-e13480-s004.pptx]

## Slide 1
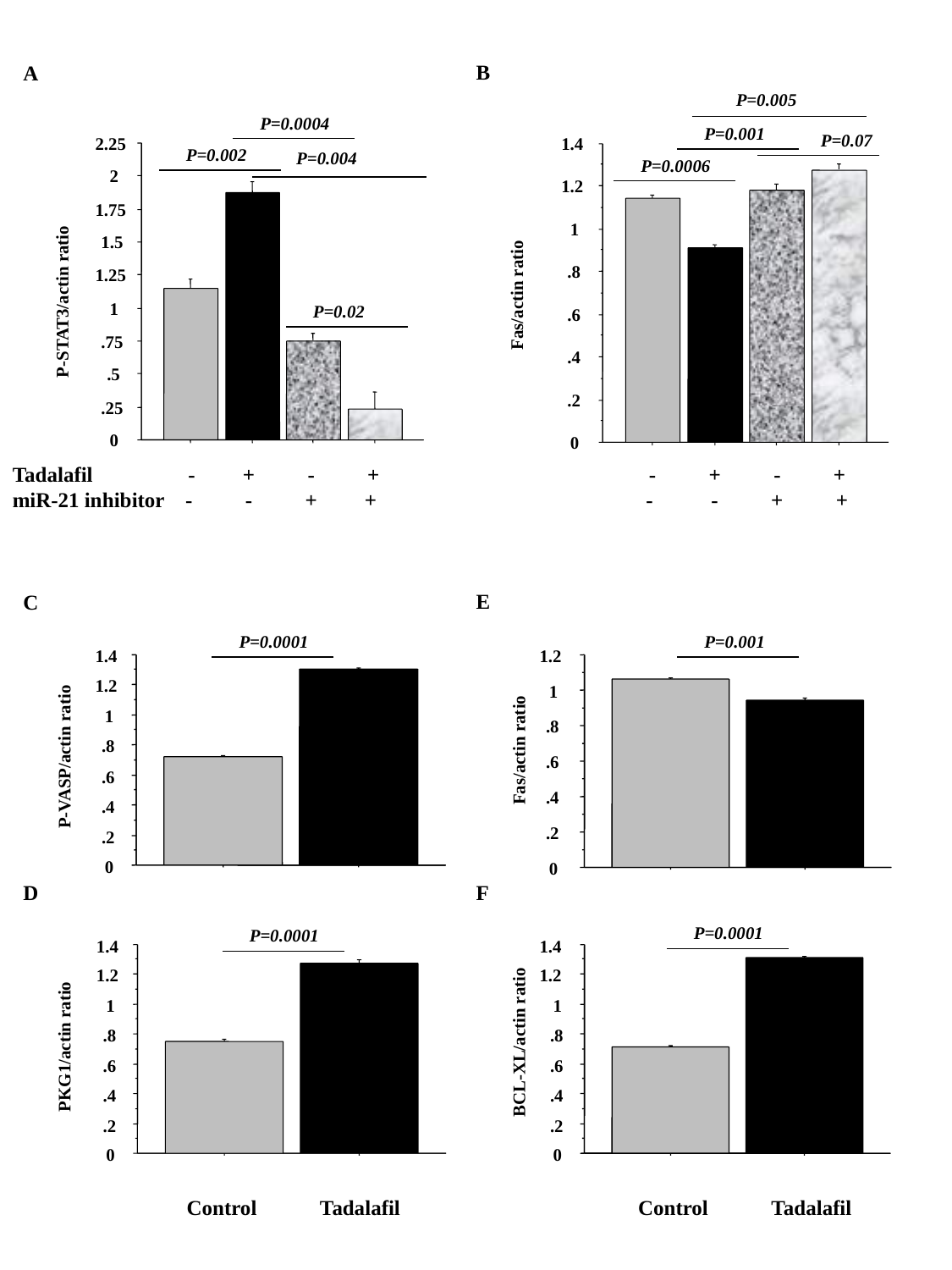

B
A
P=0.005
P=0.0004
P=0.001
P=0.07
2.25
1.4
P=0.002
P=0.004
P=0.0006
2
1.2
1.75
1
1.5
.8
1.25
Fas/actin ratio
 P-STAT3/actin ratio
P=0.02
1
.6
.75
.4
.5
.2
.25
0
0
Tadalafil - + - + - + - +
miR-21 inhibitor - - + + - - + +
E
C
P=0.0001
P=0.001
1.2
1.4
1.2
1
1
.8
.8
Fas/actin ratio
 P-VASP/actin ratio
.6
.6
.4
.4
.2
.2
0
0
F
D
P=0.0001
P=0.0001
1.4
1.4
1.2
1.2
1
1
.8
.8
BCL-XL/actin ratio
PKG1/actin ratio
.6
.6
.4
.4
.2
.2
0
0
Control Tadalafil Control Tadalafil
