# Supplementary material for: Tadalafil, a long acting phosphodiesterase inhibitor, promotes bone marrow stem cell survival and their homing into ischemic myocardium for cardiac repair
Source: Physiol Rep. 2017 Nov 15;5(21):e13480. doi: 10.14814/phy2.13480 (PMC5688776; doi:10.14814/phy2.13480)
Supplement: Supplementary file 5 — Figure S4. Tadalafil improved in vivo left ventricular (LV) function of infarcted hearts: At 7 days, iNOS (S4‐A), p‐GSKβ (S4‐B), Bcl‐xl (S4‐C), Bcl‐2 (S4‐D), total‐Akt (S4‐E), and Fas (S4‐F) expressions (western blots bands, Figure 6I) were assessed by densitometry in LV tissues extracts in control and MSCs transplanted ± tadalafil groups. [file PHY2-5-e13480-s005.pptx]

## Slide 1
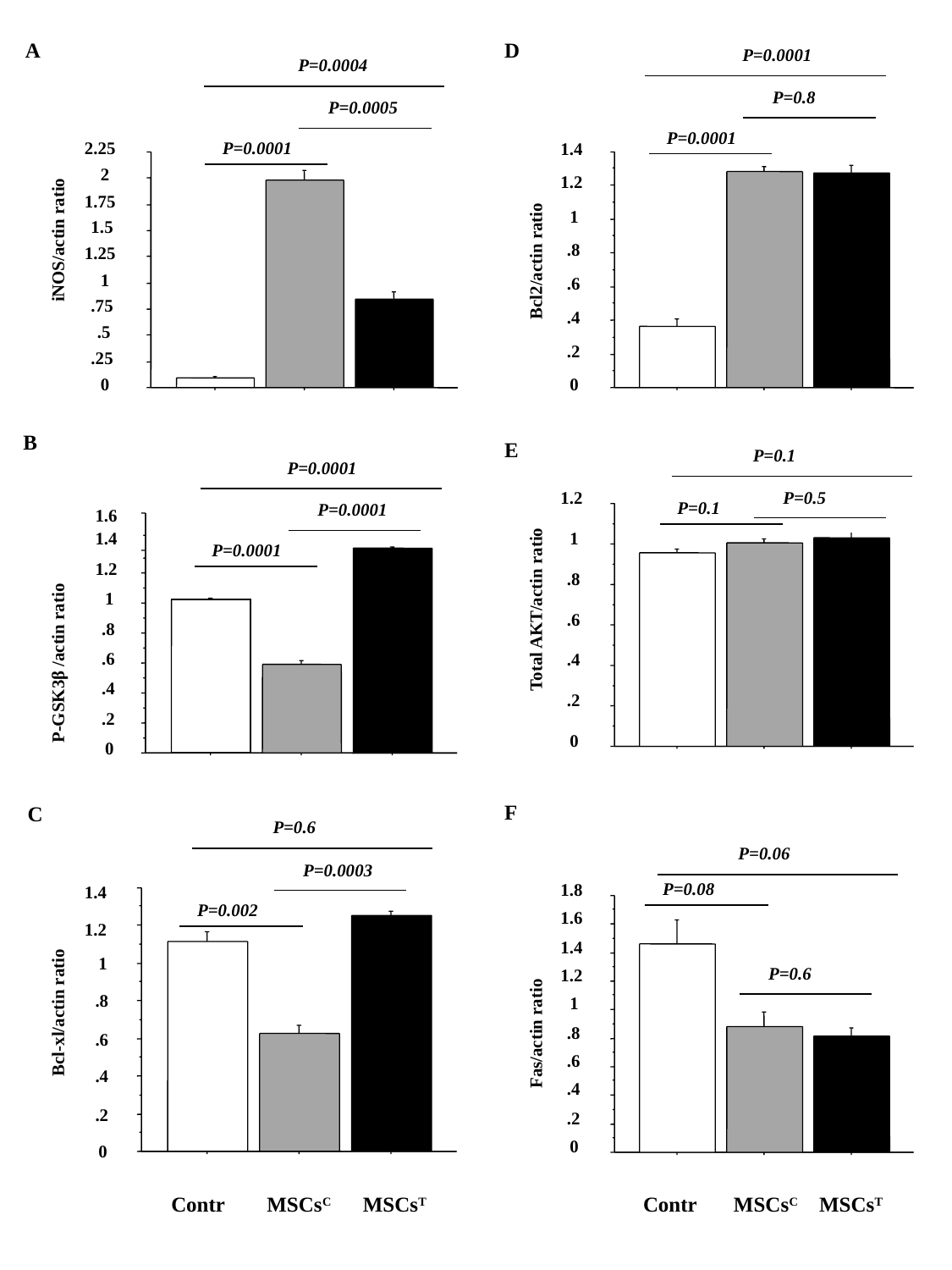

A
D
P=0.0001
P=0.0004
P=0.8
P=0.0005
P=0.0001
P=0.0001
2.25
1.4
2
1.2
1.75
1
1.5
iNOS/actin ratio
.8
1.25
Bcl2/actin ratio
1
.6
.75
.4
.5
.2
.25
0
0
B
E
P=0.1
P=0.0001
P=0.5
1.2
P=0.1
P=0.0001
1.6
1
1.4
P=0.0001
1.2
.8
1
 Total AKT/actin ratio
.6
.8
.6
.4
P-GSK3β /actin ratio
.4
.2
.2
0
0
F
C
P=0.6
P=0.06
P=0.0003
P=0.08
1.8
1.4
P=0.002
1.6
1.2
1.4
1
P=0.6
1.2
.8
1
Bcl-xl/actin ratio
Fas/actin ratio
.8
.6
.6
.4
.4
.2
.2
0
0
 Contr MSCsC MSCsT Contr MSCsC MSCsT
