# Supplementary figures and images for: Tadalafil, a long acting phosphodiesterase inhibitor, promotes bone marrow stem cell survival and their homing into ischemic myocardium for cardiac repair
Source: Physiol Rep. 2017 Nov 15;5(21):e13480. doi: 10.14814/phy2.13480 (PMC5688776; doi:10.14814/phy2.13480)

## Slide 1
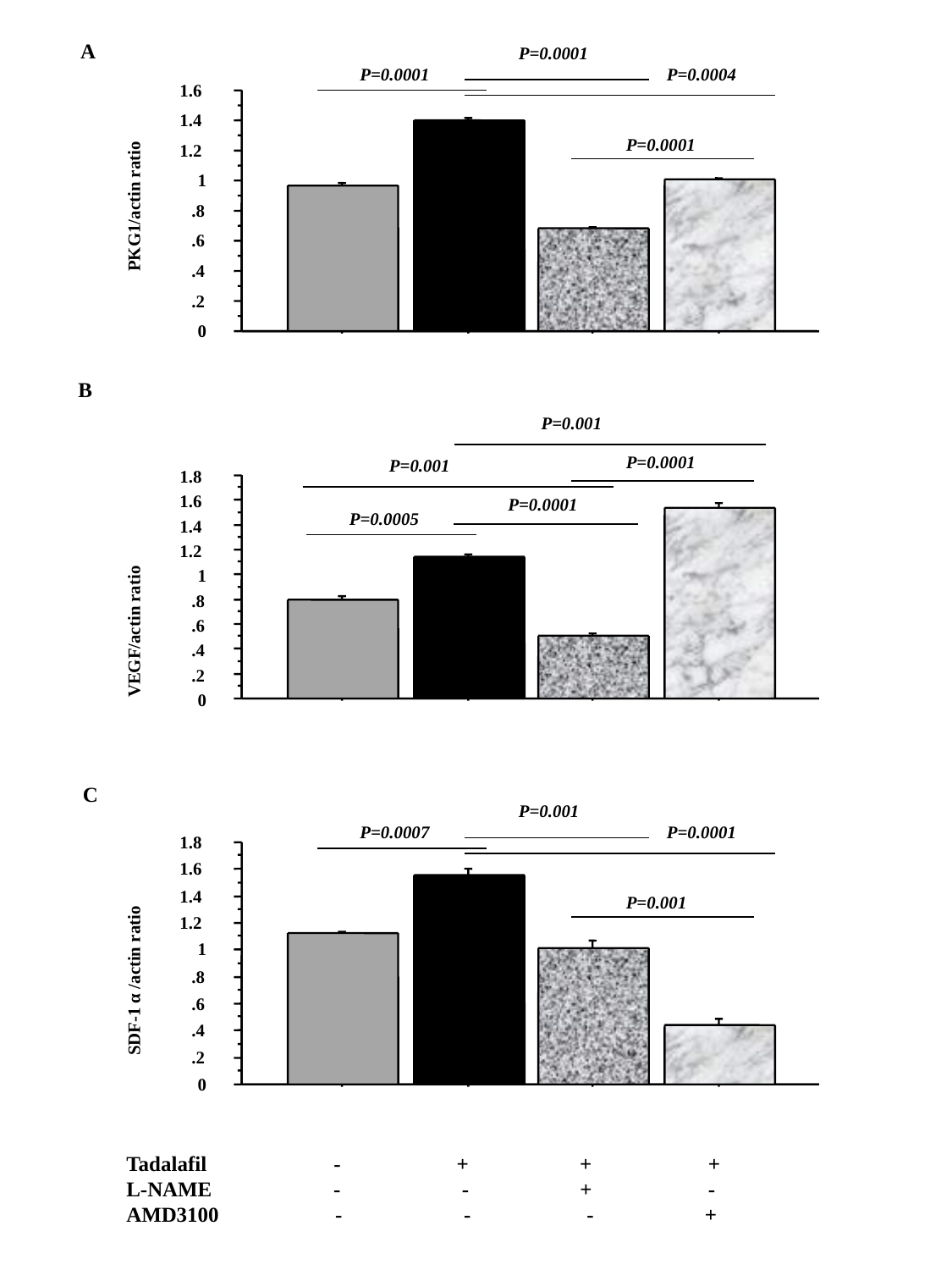

A
P=0.0001
P=0.0001
P=0.0004
1.6
1.4
P=0.0001
1.2
1
PKG1/actin ratio
.8
.6
.4
.2
0
B
P=0.001
P=0.0001
P=0.001
1.8
P=0.0001
1.6
P=0.0005
1.4
1.2
1
.8
.6
VEGF/actin ratio
.4
.2
0
C
P=0.001
P=0.0007
P=0.0001
1.8
1.6
1.4
P=0.001
1.2
1
.8
SDF-1 α /actin ratio
.6
.4
.2
0
Tadalafil - + + +
L-NAME - - + -
AMD3100 - - - +

Supplement: Supplementary file 6 — Figure S5. Tadalafil improved grafted cell survival in vivo and increased cell homing to the infarcted heart. In other groups, at 7 days, tadalafil effects on NO and CXCR4 signaling pathways were assessed after LAD ligation in 1 month post‐myeloablated rats with successful IV MSCs‐GFP+. [file PHY2-5-e13480-s006.pptx]
