# Supplementary material for: Tadalafil, a long acting phosphodiesterase inhibitor, promotes bone marrow stem cell survival and their homing into ischemic myocardium for cardiac repair
Source: Physiol Rep. 2017 Nov 15;5(21):e13480. doi: 10.14814/phy2.13480 (PMC5688776; doi:10.14814/phy2.13480)
Supplement: Supplementary file 7 — Figure S6. Proposed signaling pathways for tadalafil effect on stem cells transplantations in the infarcted myocardium. [file PHY2-5-e13480-s007.pptx]

## Slide 1
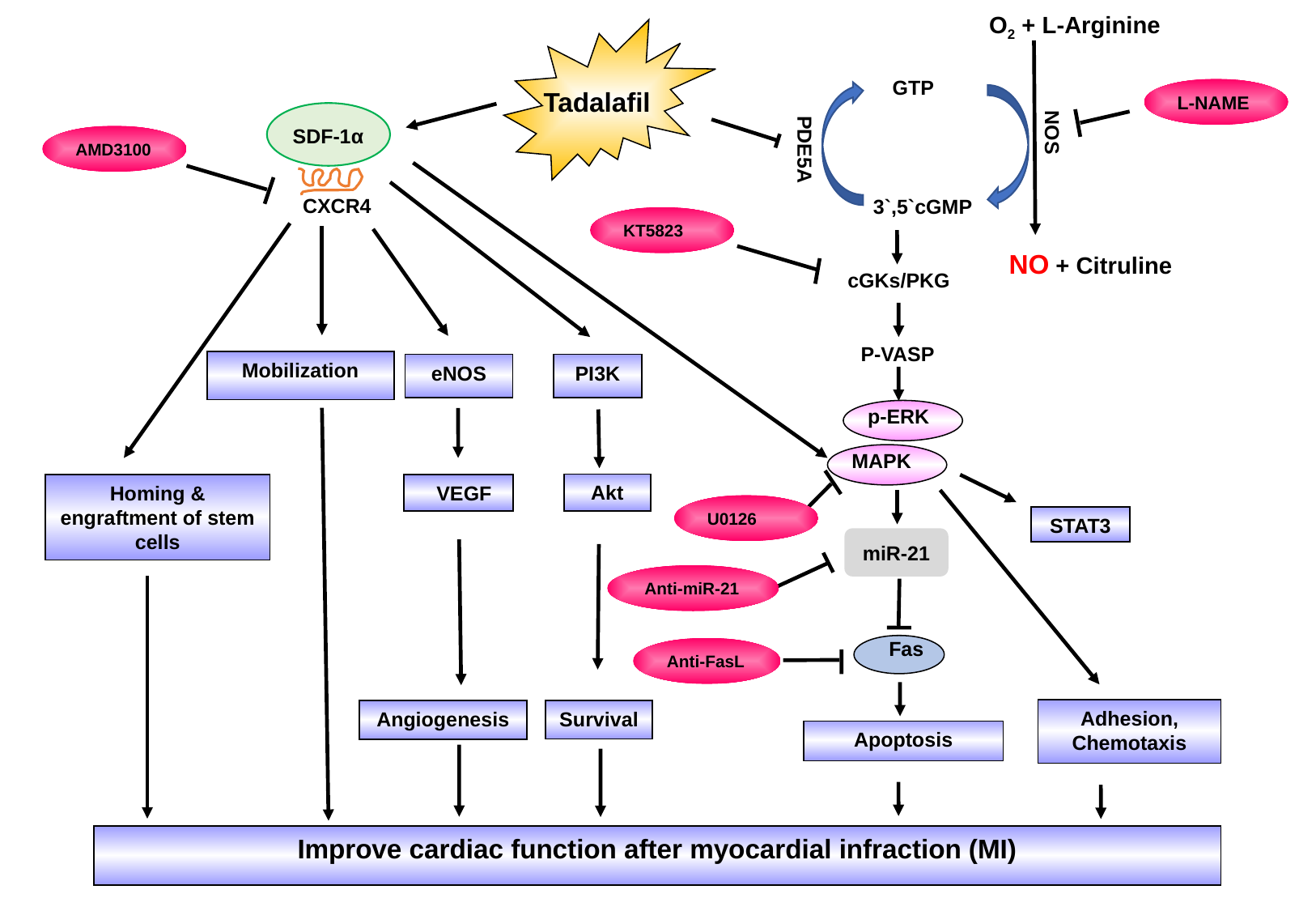

O2 + L-Arginine
Tadalafil
GTP
L-NAME
SDF-1α
CXCR4
AMD3100
NOS
PDE5A
3`,5`cGMP
KT5823
NO + Citruline
cGKs/PKG
P-VASP
Mobilization
PI3K
eNOS
p-ERK
MAPK
Akt
Homing & engraftment of stem cells
 VEGF
U0126
STAT3
miR-21
Anti-miR-21
Fas
Anti-FasL
Adhesion,
Chemotaxis
Survival
Angiogenesis
Apoptosis
Improve cardiac function after myocardial infraction (MI)
