# Supplementary material for: Tadalafil, a long acting phosphodiesterase inhibitor, promotes bone marrow stem cell survival and their homing into ischemic myocardium for cardiac repair
Source: Physiol Rep. 2017 Nov 15;5(21):e13480. doi: 10.14814/phy2.13480 (PMC5688776; doi:10.14814/phy2.13480)
Supplement: Supplementary file 8 [file PHY2-5-e13480-s008.docx]

**Supplements Figures and Legands:**

**Figure S1A.** Effect of tadalafil treatment on *in vitro* MSCs: The expression of p-STAT3 (S1-A), p-Erk1/2 (S1-B), p-Akt (S1-C), Bcl-xl (S1-D), p-VASP (S1-E), p-GSKβ (S1-F), PKG1 (S1-G) and Fas (S1-H) in MSCs extracts (western blots bands, Fig 1C) were assessed by the densitometry.

**Figure S1B.** Effect of tadalafil treatment on *in vitro* MSCs: MSCs viability (CCK-8 Assay, I) were assessed +/- tadalafil with NOS (L-NAME) or CXCR4 (AMD3100) inhibitors. Also, iNOS (S1-J), CXCR4 (S1-K) and total-Akt (S1-L) expressions (western blots bands, Figure 1F) in MSCs extracts were assessed by the densitometry in +/- tadalafil with NOS (L-NAME) or CXCR4 (AMD3100) inhibitors.

**Figure S2.** Tadalafil induced cytoprotection of *in vitro* MSCs under oxidative stress through PKG-MAPK signaling pathways: p-VASP (S2-A), PKG1 (S2-B), Fas (S2-C), BcL-xl (S2-D), p-STAT3 (S2-E) and p-Erk1/2 (S2-F) expressions (western blots bands, Fig 2D) were assessed by densitometry under oxidative stress in control and tadalafil in +/- MAPK (U0126) and PKG1 (KT5823) inhibitors.

**Figure S3.** Cytoprotective effects of tadalafil were abrogated by miR-21 inhibition in *in vitro* MSCs under oxidative stress. MSCs were transfected with pEZX-Luc vector containing Fas 3′-UTR together with a plasmid encoding miR-21; or MSCs were transfected with anti-miR-21 using siPORT^TM^ NeoFx^TM^ transfection agent. p-STAT3 (S3-A) and Fas (S3-B) expressions (western blots bands, Fig 5E) were assessed in +/- miR-21 inhibitors. Also, p-VASP (S3-C), PKG1 (S3-D), Fas (S3-E) and BcL-xl (S3-F) expressions (western blots bands, Figure 5F) were assessed by densitometry under oxidative stress in control and tadalafil in +/- FasL inhibitors.

**Figure S4.** Tadalafil improved *in vivo* left ventricular (LV) function of infarcted hearts: At 7 days, iNOS (S4-A), p-GSKβ (S4-B), Bcl-xl (S4-C), Bcl-2 (S4-D), total-Akt (S4-E) and Fas (S4-F) expressions (western blots bands, Figure 6I) were assessed by densitometry in LV tissues extracts in control and MSCs transplanted +/- tadalafil groups.

**Figure S5.** Tadalafil improved grafted cell survival *in vivo* and increased cell homing to the infarcted heart. In other groups, at 7 days, tadalafil effects on NO and CXCR4 signaling pathways were assessed after LAD ligation in 1 month post-myeloablated rats with successful IV MSCs-GFP^+^. In the LV tissue extracts, PKG1 (S5-A), VEGF (S5-B) and SDF-1α (S5-C) expressions (western blots bands, Figure 7I) were assessed by densitometry in +/- L-NAME and AMD3100.

**Figure S6.** Proposed signaling pathways for tadalafil effect on stem cells transplantations in the infarcted myocardium. Tadalafil treatment increased *ex vivo* MSCs survival via the up-regulation of miR-21-dependent suppression of Fas through the PKG-MAPK signaling pathway. Tadalafil also improved the grafted MSCs survival *in vivo* and promoted mobilization and homing of MSCs to the ischemic heart via NO/cGMP, PKG1/MAPK and SDF-1α/CXCR4 cascades. SDF-1α binds to CXCR4 receptor and modulates several biological functions through signal transduction pathways.

Abbreviation: AMD3100: CXCR4 inhibitor; ERK1/2: extracellular signal-regulated kinase 1/2; cGMP: cyclic guanosine monophosphate, Fas: CD95, tumor necrosis factor (TNF) receptor super family member; FasL: Fas ligand (CD178); KT5823: PKG1 inhibitor; L-NAME: NO synthase inhibitor; MAPK: mitogene-activated protein kinase; NO: Nitric oxide; NOS: endothelial nitric oxide synthase; PDE5A: phosphodiesterase 5A; PI3K: phosoinostol-3-phosphate kinase; PKG1: protein kinase G; SDF-1α: stromal derived factor; STAT3: signal transducers and activators of transcription-3 (phospho-STAT3); U0126: MAPK inhibitor; VEGF: vascular endothelial growth factor.
